# Physicians’ incentives, patients’ characteristics, and quality of care: a systematic experimental comparison of performance-pay systems

**DOI:** 10.1007/s10754-025-09390-x

**Published:** 2025-03-19

**Authors:** Jeannette Brosig-Koch, Mona Groß, Heike Hennig-Schmidt, Nadja Kairies-Schwarz, Daniel Wiesen

**Affiliations:** 1 Faculty of Economics and Management, Otto von Guericke University Magdeburg, Magdeburg, Germany; 2 Department of Health Care Management, University of Cologne, Cologne, Germany; 3 Department of Economics, University of Bonn, Bonn, Germany; 4 Institute for Health Services Research and Health Economics, Medical Faculty and German Diabetes Center Düsseldorf, Heinrich Heine University Düsseldorf, Düsseldorf, Germany; 5 Erasmus School of Health Policy & Management (ESHPM), Erasmus University of Rotterdam, Rotterdam, The Netherlands; 6 Health Economics Research Center CINCH, University of Duisburg-Essen, Essen, Germany

**Keywords:** Pay for performance, Fee-for-service, Capitation, Treatment quality, Heterogeneous patients, Laboratory experiment, C91, C93, I11

## Abstract

How performance pay affects physicians’ medical service provision and the quality of care is relevant for researchers and policy-makers alike. This paper systematically studies how performance pay, complementing either fee-for-service or capitation, affects physicians’ medical service provision and the quality of care for heterogeneous patients. Using a series of controlled behavioral experiments with physicians and students, we test the incentive effect of performance pay at a within-subject level. We consider a performance pay scheme which grants a discrete bonus if a quality threshold is reached, which varies with the patients’ severity of illness. We find that performance pay significantly reduces non-optimal service provision and enhances the quality of care. Effect sizes depend on the patients’ severity of illness and whether performance pay is blended with fee-for-service or capitation. Health policy implications, including a cost benefit analysis of introducing performance pay, are discussed.

**Supplementary Information:**

The online version contains supplementary material available at 10.1007/s10754-025-09390-x.

## Introduction

Paying physicians for performance has become prominent among health-policy makers around the world, for example, in high-income countries such as the USA (e.g., Rosenthal et al., 2006, Song et al., 2019), the United Kingdom (e.g., Doran et al., 2006, Roland and Campbell, 2014), and in low and middle income countries (e.g., Sherry et al., 2017, Celhay et al., 2019, Kovacs et al., 2020, Diaconu et al., 2021). This seems natural, as the general idea is appealing: Performance pay (P4P) incentivizes healthcare providers to enhance the quality of care as a payment is usually granted if a quality threshold is reached. More traditional physician payment systems such as capitation (CAP) or fee-for-service (FFS), which had been commonly implemented, do not inhibit explicit incentives to improve quality. FFS incentivizes physicians to overserve patients, whereas CAP embeds an incentive to underserve them. Thus, paying physicians on the basis of direct performance measures has attracted particular attention.

In practice, P4P is typically blended with either FFS or CAP. The incentives of the two P4P-systems are diametrically opposed: While FFS$+$P4P implies an incentive to reduce oversupply of medical services, CAP$+$P4P incentivizes physicians to enhance medical service provision. In the empirical literature, a systematic comparison of the effectiveness of these blended P4P-systems is still lacking. It thus remains not well understood how patients with different severities of illness are affected by incentives of the P4P-systems. The heterogeneous impact of payment incentives on different patient types has been indicated in recent empirical and experimental studies (e.g., Clemens and Gottlieb, 2014, Brosig-Koch et al., 2017a).

Despite the wide range of employed P4P schemes, the evidence base supporting their effective use is evolving slowly (Wilding et al., 2022) and, if at all, the empirical studies on how P4P affects the quantity and the quality of care yield mixed results (Scott et al., 2011; Emmert et al., 2012; Eijkenaar et al., 2013; Milstein & Schreyögg, 2016; Scott et al., 2018; Cattel & Eijkenaar, 2020; Jia et al., 2021). Potential reasons for the difficulty in establishing a causal link between performance pay and physicians’ provision behavior comprise the likely endogeneity of institutions (e.g., Baicker and Goldman, 2011), biased performance measures (e.g., Mullen et al., 2010), measurement errors (e.g., Campbell et al., 2009), limited availability of data (e.g., Gravelle et al., 2010), and the simultaneous introduction of P4P with other interventions (e.g., Lindenauer et al., 2007). Moreover, the design of a P4P-system seems key to effectively change physician behavior (e.g., Epstein, 2012, Maynard, 2012, Kristensen et al., 2016, Anselmi et al., 2020).

Our study aims to contribute to a better understanding of P4P-effects on the quantity and quality of care. We implemented a controlled behavioral experiment, in which physicians’ financial incentives under FFS and CAP are mirror images of each other. FFS and CAP are complemented with performance-based bonuses paid whenever a quality threshold tied to a patient’s optimal health outcome (e.g., services according to guidelines) is reached. Meeting the threshold still allows for non-optimal service provision, due to the assumed asymmetric information between physician and payer. Such a service provision might thus increase the physician’s profit while at the same time not rendering optimal care. The patient population is kept constant: All physicians see the same patients with heterogeneous severities of illness and marginal health-benefits twice, in the basic and in the respective performance-pay conditions. The constant patient population and the mirrored, symmetric incentives allow us to systematically compare the blended P4P-schemes P4P$+$FFS and P4P$+$CAP, an analysis missing in the literature so far.

We conducted the incentivized experiments with physicians via labs-in-the-field and with students in the lab. To establish the causal link between P4P and quantity and quality of care, we exogenously vary physicians’ payment at a within-subject level from the baseline non-blended payments to blended performance-pay systems. In a medically framed task, subjects decide on the quantity of medical services for a series of stylized patients varying in their severities of illness and marginal health-benefits. Quantity choices determine the physician’s profit and the patient’s health-benefits, which is measured in monetary terms. Patients’ health-benefits are made salient in that subjects’ decisions in the lab affect real-world patients’ health. The money corresponding to the aggregated health-benefits is transferred to a charity and is used exclusively for surgery of cataract patients.

Using a behavioral experiment, we analyze, first, how P4P affects medical service provision and the quality of care when complementing FFS. Second, we study whether the P4P-effect depends on patients’ severities of illness and marginal health-benefits. Third, we analyze the P4P-effect if blended with CAP, and fourth, we investigate effects for heterogeneous patients. Finally, we compare whether P4P-effects vary between FFS+P4P and CAP+P4P, despite their mirror-image design of incentives.

Behavioral results indicate that P4P reduces non-optimal service provision and enhances the quality of care compared to FFS and CAP. However, the P4P-effects significantly vary with patients’ severities. Under FFS, the marginal effect of P4P on the quantity and quality of care decreases in the patients’ severity of illness. Under CAP, we observe the reverse pattern. Behavioral results thus indicate that a threshold-based P4P bonus adjusted to the patient severity, a concept similar to diagnosis-related groups in hospital payment systems, is most beneficial for mildly-ill patients under FFS, whereas it is most beneficial for severely-ill patients under CAP. Patients with an intermediate severity of illness are almost equally treated under both performance-pay systems. While our results suggest that P4P seems to be an effective means to counteract non-optimal service provision under FFS and CAP, they also emphasize the importance of the payment system’s design. Based on these findings, cost-benefit analyses within the confines of the symmetric mirror-image design imply that the financial resources needed to induce a one-unit increase in patient health-benefit through physicians’ service provision vary between performance-pay systems.

We contribute to several streams in the health economics literature. First, we complement empirical studies evaluating P4P programs, which in many cases rely on aggregated, longitudinal data. The empirical evidence for a P4P-effect is rather mixed in primary care (e.g., Scott et al., 2011), outpatient care (e.g., Jia et al., 2021), and inpatient care (e.g., Mathes et al., 2019). Using longitudinal data, Mullen et al. (2010) find a small positive effect of P4P on process quality of multi-specialty medical groups. Studies mostly evidence some increase in a few clinical processes; yet, the P4P-effects on outcome quality are not clear (e.g., Li et al., 2014). While empirical studies often rely on aggregated data, we add insights on a causal effect of P4P at the individual subject level. The only empirical study analyzing individual within-provider changes is Cadena and Smith (2022), who report moderate increases in productivity due to P4P. The highly controlled environment in our experiment allows us to implement ‘clean’ measures for the quality of care at the individual physician level and to analyze the incentive effect of P4P under patient heterogeneity.

Second, our study contributes to the scarce experimental literature analyzing physician performance pay. These studies provide first evidence for a positive effect of P4P on treatment behavior of practicing physicians, prospective physicians (medical students) and non-medical students in the role of physicians. Cox et al. (2016b) report that P4P incentivizes cost-effective reductions in hospital re-admissions in a lab experiment with US students. Using a simulation-based randomized controlled trial, Green et al. (2020) emphasize the importance of heterogeneous patients. Brosig-Koch et al. (2022) study the effectiveness of bonus payments for information provision in patient referrals with a sample of German students. Brosig-Koch et al. (2024) analyze the behavior of a representative primary-care physician sample in a threshold-based P4P-system, analogous to the CAP+P4P condition in this paper. Oxholm et al. (2021) find P4P to affect the allocation of medical care across patients with different responsiveness to treatment compared to CAP-payments in a sample of Danish medical students. Our study differs, however, from earlier studies in that we systematically compare the effects of FFS *and* CAP blended with P4P accounting for heterogeneous patients. We also analyze the cost and benefits of introducing P4P.

Finally, taking a broader perspective, we also relate to behavioral experiments in health (Galizzi and Wiesen 2017; 2018) analyzing physician payment systems (Hennig-Schmidt et al., 2011; Hennig-Schmidt & Wiesen, 2014; Green, 2014; Brosig-Koch et al., 2016; Bejarano et al., 2017; Lagarde & Blaauw, 2017; Brosig-Koch et al., 2017a, 2024; Di Guida et al., 2019; Martinsson & Persson, 2019; Reif et al., 2020; Green & Kloosterman, 2022). More generally, we also add to experimental studies on credence goods markets for which healthcare is a key example (e.g., Dulleck and Kerschbamer, 2006; Dulleck et al., 2011; Angerer et al., 2023). Due to information asymmetries between experts (physicians) and customers (patients), there is a high potential to exploit patients, for example, through overtreatment under FFS. In our experiment, we assume patients to be passive and fully insured (McGuire, 2000) accepting medical service provision of subjects in the role of physicians. Neutrally-framed credence-goods experiments showed that overtreatment can be reduced by second opinions (Mimra et al., 2016), competition (Huck et al., 2016), separating treatment and diagnosis decisions (Greiner et al., 2017), and monitoring (Hennig-Schmidt et al., 2019, Angerer et al., 2021, Groß et al., 2021). We complement these experiments by investigating whether performance-based payments, which implicitly rely on monitoring physicians’ performance, are a useful means to reduce overtreatment, for example, under FFS.

## Methods: experimental design and hypotheses

### Decision situation

In our medically-framed experiment, subjects decide in the role of physicians on medical care provision. Using a within-subject design, all subjects decide under non-blended and blended payment systems. First, subjects are incentivized by a baseline payment being FFS or CAP. Second, we introduce P4P in addition to the respective baseline payments (FFS+P4P or CAP+P4P). We randomly assign subjects to one of the two conditions.

More formally, subjects in the role of physicians decide on the quantity of medical services $q\in [0,10]$ for nine different patients, who have different illnesses $k\in \left\{ A,B,C\right\}$ and severities of illness $l\in \left\{ x,y,z \right\}$. Physicians’ payment is $R(q)= L + pq + b_{l} I_{b_l}$; *L* is the lump-sum, *p* the fee per service, and $b_l$ the bonus payment. $I_{b_l}$ denotes an indicator variable which equals 1, if the quality threshold $q\in |q-q^*|\le 1$, with $q^*$ being the patient-optimal care, is met, and 0 otherwise. In FFS, $L=0$ and $b_l^{\hbox {\tiny FFS}}=0$. In CAP, $p=0$, and $b_l^{\hbox {\tiny CAP}}=0$. A physician profit is given as $\pi (q) = L + pq + b_l I_{b_l}-c(q)$, with $L, p, b_l\ge 0$, $c(q)=q^2/10$, $c'(q)>0$ and $c''(q)>0$. For each patient, the subject in the role of a physician simultaneously determines her profit $\pi (q)$ and the patient’s health-benefit *H*(*q*). The health-benefit function is: $H(q) = H_0+\theta q$ if $q\le q^*$ and $H_1-\theta q$ if $q \ge q^*$, with $H_0, H_1 \ge 0$, $\theta>0$, and with a global optimum at $q^*$ on $q \in (0,10)$. For illnesses *A* and *B* $\theta = 1$, and for illness *C* $\theta = 2$. For illnesses *A*, *B*, and *C*, the maximum health-benefit is $H_{A} (q^*)=7$, $H_{B} (q^*)=10$, and $H_{C} (q^*)=14$, respectively.^1^ Patient-optimal care $q^*$ depends on the severity of illness *l*. For mildly (*x*), intermediately (*y*), and highly (*z*) severe illnesses, the patient-optimal quantities are $q_x^*=3$, $q_y^*=5$, and $q_z^*=7$, respectively; see Figure A.2 for an illustration.^2^ All experimental parameters are common knowledge. Subjects are aware of cost, payment, profit, and the patient’s health-benefit for each quantity; see instructions in Online-Appendix A.3.

Although the decision situation abstracts from the complexity of everyday medical practice, it was validated in interviews with practicing physicians and leading experts in physician remuneration in Germany. They confirmed that the design captures the essential characteristics of treatment decisions that physicians face in their daily practice. Moreover, ex-post experimental questionnaire data from general practitioners also confirms this view (Brosig-Koch et al., 2024).^3^

### Payment systems

Table 1 provides an overview of the payment systems. In FFS, subjects in the role of physicians receive a fee ($p=2$) per service. Under CAP, they get a lump sum $L=10$ per patient. The maximum attainable profit is thus 10 in both payment systems. The profit-maximizing quantity of medical services for each of the nine patients is $\hat{q}^{\hbox {\tiny FFS}}_j=10$ and $\hat{q}^{\hbox {\tiny CAP}}_j=0$ in FFS and CAP, respectively. This reflects the prevalent incentives for overprovision under FFS and underprovision under CAP. While varying the design components of the payment systems, we keep maximum profit levels and marginal profits constant; see Figure A.3 in Online-Appendix A.2.

**Table 1:** Payment parameters

First part of the experiment					Second part of the experiment						Subjects (physicians, medical
(Non-blended payment systems)					(Blended payment systems)						students, non-medical students)
Payment	*L*	*p*	*R*		Payment	Severity *l*	*L*	*p*	$b_l$	*R*	
FFS	–	2	2*q*		FFS$+$P4P	*x*	–	2	5.6	$2q+5.6$	52 (10, 22, 20)
						*y*	–	2	3.6	$2q+3.6$	
						*z*	–	2	2.4	$2q+2.4$	
CAP	10	–	10		CAP$+$P4P	*x*	10	–	2.4	$10 + 2.4$	55 (10, 22, 23)
						*y*	10	–	3.6	$10 + 3.6$	
						*z*	10	–	5.6	$10 + 5.6$	

This table shows the parameters and the number of participants in each experimental part. Data for the non-blended payment systems correspond to a part of the data analyzed in Brosig-Koch et al. (2016)

P4P is granted if the provided quantity of care does not deviate by more than one unit from the patient-optimal quantity ($q^*$; $|q-q^*|\le 1$). We thereby assume that the quality is not fully contractible due to information asymmetry. P4P thus mitigates incentives to overserve under FFS and to underserve under CAP. In our experiment, profit-maximizing quantities under P4P are more aligned with the patient-optimal quantities compared to non-blended FFS or CAP, but do not coincide with them. We are thus able to differentiate between profit maximization and patient-optimal care in our P4P conditions.

Rates of the discrete bonus are set such that incentives are comparable across payment systems. The bonus implies an increase in the maximum profit $\pi (\hat{q}_j)$ by 20 percent. For severities *x*, *y*, and *z*, $b_x^{\hbox {\tiny FFS}}=5.6$, $b_y^{\hbox {\tiny FFS}}=3.6$, $b_z^{\hbox {\tiny FFS}} =2.4$ in FFS$+$P4P, and $b_x^{\hbox {\tiny CAP}}=2.4$, $b_y^{\hbox {\tiny CAP}}=3.6$, $b_z^{\hbox {\tiny CAP}} =5.6$ in CAP$+$P4P, respectively. For each severity, choosing $\hat{q}_j$ equal to 4, 6, or 8 (2, 4, or 6) in FFS$+$P4P (CAP$+$P4P) thus yields a profit of 12 for the subject in the role of a physician.

### Procedure

Overall, 107 subjects participated in our experiment. Among these were 44 medical and 43 non-medical students who took part in the lab experiments, and 20 physicians who participated in the lab-in-the-field experiments. Each subject was randomly assigned to only one of the two baseline payment systems. 55 subjects took part in CAP/CAP$+$P4P and 52 in FFS/FFS$+$P4P, with 22 medical students and 10 physicians under each payment system.

The computerized experiment was programmed with z-Tree (Fischbacher, 2007). Physicians and students were presented with identical computer screens, instructions, and comprehension questions. The main difference was a higher exchange factor from the experimental currency to Euro for physicians compared to students.^4^ The lab-in-the-field experiments were conducted with the mobile lab of the Essen Laboratory for Experimental Economics (elfe) at the Academy for Training and Education of Physicians in Bad Nauheim (Germany) in 2012 and 2013. At the Academy, physicians were recruited by announcements in their training courses, and they voluntarily participated after their courses. The lab experiments were conducted between 2011 and 2013 at elfe at the University of Duisburg-Essen. Student subjects were recruited online via ORSEE (Greiner, 2015). For more details, see Appendix A.1.

### Behavioral hypotheses

To derive behavioral hypotheses, we assume that a physician derives utility from own profit and the patient’s health-benefit. The weight the physician attaches to the patient’s health-benefit is interpreted as a measure for physician altruism (e.g., Ellis and McGuire, 1986, Kolstad, 2013). For an illustrative model, which is the basis for the hypotheses below, see Online-Appendix B.

First, we consider a physician’s behavior under FFS and CAP. For the profit- and patient-benefit parameters in our experiment and a given level of physician altruism, we conjecture that FFS induces overprovision, which decreases in the patient’s severity of illness and in the marginal health-benefit. On the contrary, we expect CAP to incentivize underprovision of care, which increases in the severity of illness and decreases in the marginal health-benefit. Ample evidence for these conjectures exists from related experiments (e.g., Hennig-Schmidt et al., 2011, Brosig-Koch et al., 2016; 2017a, Martinsson and Persson, 2019, Brosig-Koch et al., 2024). Severity-of-illness related heterogeneous behaviors are particularly relevant in our experiment, as the levels of P4P are tied to the patients’ severity of illness; for an illustration, see Figure A.3 in Online-Appendix A.2.

When introducing P4P the bonus $b_{l}$ is granted if and only if a physician’s treatment decision meets the quality threshold $|q-q^*|\le 1$. Quality is not fully contractible. By linking performance pay to patient-optimal care, the interests of the physician and the patient become more aligned. While P4P incentivizes less altruistic physicians to provide medical services ‘close’ to the patient-optimal quantity, incentives for underprovision under CAP and overprovision under FFS are still inherent albeit to a substantially lower extent. Hence, we hypothesize that P4P reduces overprovision of medical services in FFS and underprovision in CAP. We state the following hypotheses on the effects of threshold-based performance-pay systems with discrete bonuses:

#### Hypothesis 1

(**FFS**$+$**P4P**) Performance pay reduces the overprovision of medical services under fee-for-service and increases the quality of care.

#### Hypothesis 2

(**FFS**$+$**P4P and patients’ characteristics**) Under performance pay and fee-for-service, the performance-pay effect on medical service provision and the quality of care decreases in the patient’s severity of illness and the marginal health-benefit.

#### Hypothesis 3

(**CAP**$+$**P4P**) Performance pay reduces the underprovision of medical services under capitation and enhances the quality of care.

#### Hypothesis 4

(**CAP**$+$**P4P and patients’ characteristics**) Under performance pay and capitation, the performance-pay effect increases in the patient’s severity of illness and the marginal health-benefit.

Following directly from Hypotheses 2 and 4, we state our main hypothesis on the comparison between performance-pay systems:

#### Hypothesis 5

(**FFS**$+$**P4P and CAP**$+$**P4P**) FFS$+$P4P leads to a larger improvement in the quality of care for mildly-ill patients compared to CAP$+$P4P. For severely-ill patients, the increase in quality of care is larger for CAP$+$P4P, while for intermediately-ill patients, the quality of care does not differ between the two pay-for-performance systems.

To test our hypotheses, we analyze – besides the quantity of medical services – the quality of care by two measures: a choice-based measure capturing the absolute deviation from the patient-optimal quantity $\rho =|q-q^*|$ and an outcome-based measure capturing the proportional patient health-benefit. The latter is defined by $\tilde{H}_{kl}=\frac{H^{\min }_{kl}-H_{kl}}{H^{\min }_{kl}-H^*_l}$, with $H_{kl}$ being the health-benefit determined by the physician quantity choice, $H^*_{l}$ being the maximum and $H^{\min }_{kl}$ the minimum health-benefit for patient *kl*.

## Results

### Descriptive analyses

**Fig. 1:**
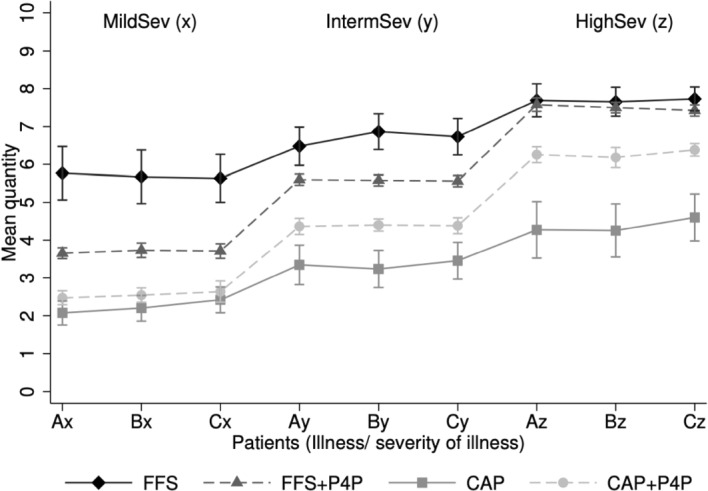
Mean quantity by patients’ health characteristics. *Notes.* This figure shows the mean quantity with 95% confidence interval under the four payment systems for each of the nine patients *kl*. Patients vary by their illness $k={A, B, C}$ and severity of illnesses *l* with mild (*x*), intermediate (*y*), and high (*z*) severities of illnesses

Figure 1 illustrates the average *quantity* of medical services for the four payment systems; see also Figure C.1 in Online-Appendix C. We find that, on average, subjects provide significantly more services under FFS (mean 6.69, s.d. 2.07) than under CAP (mean 3.32, s.d. 2.13), ($p <0.001$, two-sided Mann-Whitney U-test). This finding is in line with earlier experimental studies (Hennig-Schmidt et al., 2011; Hennig-Schmidt & Wiesen, 2014; Green, 2014; Brosig-Koch et al., 2016, 2017a; Martinsson & Persson, 2019). In FFS$+$P4P, quantities of medical services decrease by about 16.4 percentage points (mean 5.59, s.d. 1.71), and in CAP$+$P4P, they increase by about 32.5 percentage points (mean 4.40, s.d. 1.66); see Table C.1 in Appendix C.

Concerning the *quality* of care, the average deviation from the patient-optimal quantity $\rho$ is 1.82 (s.d. 1.95) under FFS. P4P reduces the average non-optimal service provision $\rho$ to 0.63 (s.d. 0.55), which is a reduction by 65.4 percentage points. Under CAP, $\rho =$ 1.77 (s.d. 2.01), while under CAP+P4P $\rho$ declines to 0.65 (s.d. 0.75), a decrease by 63.3 percentage points; see Table C.1 in Online-Appendix C. The proportional health-benefit $\tilde{H}$, realizes, on average, around 71% of the maximum health-benefit in the non-blended payment schemes and around 90% in the blended payment systems. The P4P-effect thus corresponds to an overall increase in the proportional health-benefit by 19 percentage points under CAP+P4P and FFS+P4P; see Table C.1 in Appendix C. On the aggregate, P4P leads to a significant increase in both the choice-based and the outcome-based quality measures ($p<0.001$, Wilcoxon signed-rank test, two-sided).

Further, patients’ severity of illness substantially affects subjects’ behavior in all payment systems. Overprovision of medical services is highest for mildly-ill patients in both FFS conditions, and underprovison is highest for severely-ill patients in both CAP conditions. The behavioral effect is rather less pronounced for the marginal health-benefit; see Table C.1 in Online-Appendix C.

### Effects of performance pay blended with fee-for-service

To estimate the P4P-effect, we use OLS-regressions for the independent variables $q_{ij}$ (quantity chosen), and $\rho _{ij} =|q_{ij}-q^*_{j}|$ (absolute deviation from optimal care). We use a fractional probit response model for $\tilde{H}_{ij}$ (proportional health-benefit), scaled between 0 and 1. Our base econometric specification is as follows:

1 $$\begin{aligned} y_{ij}= & \alpha + \beta _1\hbox {P4P} + \beta _2{\textsc {IntermSev}} + \beta _3{\textsc {HighSev}} + \beta _4{\textsc {HighMHB}} + \beta _5\textbf{X}_i + \epsilon _{ij}. \end{aligned}$$

IntermSev and HighSev are dummy variables for intermediate and high severities of illness, respectively. HighMHB is a dummy for the marginal health-benefit being 1 if $\theta =2$ (high), and 0 otherwise ($\theta =1$, low). P4P is a dummy variable indicating the introduction of P4P. $X_{i}$ is a vector of subject *i*’s characteristics comprising gender, personality traits, and subjects’ medical background (non-medical students, medical students or physicians). We account for potentially confounding effects by medical background as previous experimental evidence indicates that the intensity of responses to incentives might differ across subject pools (e.g., Hennig-Schmidt and Wiesen, 2014, Brosig-Koch et al., 2016, Reif et al., 2020). Our estimated P4P-effects remain stable, however, when we control for subjects’ medical background and other characteristics; see Tables C.4 to C.7 in Appendix C.^5^ Estimation results show that overprovision is reduced and quality of care increases, when FFS is blended with P4P; see Models (1), (4), and (7) in Table 2. Supporting Hypothesis 1, FFS$+$P4P leads to a highly significant reduction in quantity by, on average, 1.10 medical services. Non-optimal care also declines highly significantly, by 1.20 medical services on average. The proportional health-benefit increases by about 18.9 percentage points under P4P$+$FFS. We summarize as follows:

#### Result 1

(**FFS**$+$**P4P**) *Complementing fee-for-service with a threshold-based performance-pay system leads to a decrease in overprovision of medical services, which corresponds to an increase in the quality of medical care and in the proportional health-benefit.*

**Table 2:** Regression models on the effect on quantity and quality under FFS conditions

	A. Quantity of medical services *q*			B. Absolute deviation from optimal care $\rho$			C. Proportional health-benefit $\tilde{H}$		
Method:	OLS	OLS	OLS	OLS	OLS	OLS	Frac. Probit	Frac. Probit	Frac. Probit
Model:	(1)	(2)	(3)	(4)	(5)	(6)	(7)	(8)	(9)
P4P	-1.100$$^{***}$$			-1.199$$^{***}$$			0.189$$^{***}$$		
	(0.185)			(0.172)			(0.025)		
IntermSev	1.439$$^{***}$$	1.000$$^{***}$$	1.439$$^{***}$$	-0.529$$^{***}$$	-0.936$$^{***}$$	-0.529$$^{***}$$	0.004	0.019	0.004
	(0.086)	(0.147)	(0.086)	(0.086)	(0.149)	(0.086)	(0.010)	(0.012)	(0.010)
HighSev	2.901$$^{***}$$	2.000$$^{***}$$	2.901$$^{***}$$	-0.997$$^{***}$$	-1.782$$^{***}$$	0.997$$^{***}$$	0.133$$^{***}$$	0.184$$^{***}$$	0.133$$^{***}$$
	(0.129)	(0.230)	(0.129)	(0.141)	(0.256)	(0.141)	(0.016)	(0.023)	(0.016)
HighMHB	-0.016	-0.016	0.010	-0.054	-0.054	-0.074	0.009	0.009	0.008
	(0.053)	(0.053)	(0.089)	(0.051)	(0.051)	(0.087)	(0.008)	(0.008)	(0.011)
P4P$$\times $$ MildSev		-1.994$$^{***}$$			-1.994$$^{***}$$			0.187$$^{***}$$	
		(0.277)			(0.277)			(0.021)	
P4P$$\times $$ IntermSev		-1.115$$^{***}$$			-1.179$$^{***}$$			0.162$$^{***}$$	
		(0.192)			(0.183)			(0.021)	
P4P$$\times $$ HighSev		-0.192			-0.423$$^{***}$$			0.079$$^{***}$$	
		(0.132)			(0.111)			(0.017)	
P4P$$\times $$ LowMHB			-1.083$$^{***}$$			-1.212$$^{***}$$			0.171$$^{***}$$
			(0.195)			(0.179)			(0.022)
P4P$$\times $$ HighMHB			-1.135$$^{***}$$			-1.173$$^{***}$$			0.153$$^{***}$$
			(0.177)			(0.172)			(0.018)
Constant	5.623$$^{***}$$	6.070$$^{***}$$	5.615$$^{***}$$	2.621$$^{***}$$	3.019$$^{***}$$	2.627$$^{***}$$			
	(0.315)	(0.350)	(0.318)	(0.315)	(0.354)	(0.317)			
Wald test (*p*-value)									
$H_0$: P4P$\times$ MildSev $=$ P4P$\times$ IntermSev		<0.001			<0.001			0.010	
$H_0$: P4P$\times$ MildSev$=$ P4P$\times$ HighSev		<0.001			<0.001			<0.001	
$H_0$: P4P$\times$ IntermSev$=$ P4P$\times$ HighSev		<0.001			<0.001			<0.001	
$H_0$: P4P$\times$ LowMHB$=$ P4P$\times$ HighMHB			0.556			0.658			0.062
Observations	936	936	936	936	936	936	936	936	936
Subjects	52	52	52	52	52	52	52	52	52
(Pseudo) $R^{2}$	0.563	0.599	0.563	0.336	0.379	0.336	0.150	0.157	0.150

This table shows parameter estimates from OLS regressions (Panel A and B) and average marginal effects from fractional probit response regressions (Panel C). Robust standard errors clustered for subjects are shown in parentheses. P4P is a dummy variable indicating the introduction of P4P. IntermSev and HighSev are dummy variables for intermediate and high severities of illness. HighMHB is a dummy for the marginal health-benefit being 1 if $\theta = 2$ (high), and 0 otherwise ($\theta = 1$, low). All models control for individual characteristics which comprise gender, medical background (non-medical student, medical student, physician), and personality traits; for the respective estimates, see Table C.5 in Online-Appendix C. * $p<0.10$, ** $p<0.05$, and, *** $p<0.01$

Before testing Hypothesis 2, we analyze how patients’ characteristics affect the decisions of subjects in the role of physicians and the quality of care under non-blended FFS. Compared to mildly-ill patients, treatment quantities increase significantly for intermediately and severely-ill patients by, on average, 1.44 and 2.90 medical services, respectively; see Model (1) of Table 2. These findings are in line with earlier experiments (e.g., Brosig-Koch et al., 2017a, Martinsson and Persson, 2019). Considering quality, non-optimal care significantly decreases with increasing severity (Model (4)), and the proportional health-benefit increase for severely-ill patients is significantly higher than for mildly-ill patients (13.3 percentage points), but does not significantly differ between intermediately and mildly-ill patients (Model (7)).

To estimate the moderating effects of patients’ severities of illness on responses to P4P, we consider the following model:

2 $$\begin{aligned} y_{ij}= & \alpha + \beta _1{\textsc {IntermSev}} + \beta _2{\textsc {HighSev}} + \beta _3{\textsc {HighMHB}} + \beta _4 {\textsc {P4P}} \textbf{x} {\textsc {MildSev}} \nonumber \\ & + \beta _5 {\textsc {P4P}}\textbf{x} {\textsc {IntermSev}} + \beta _6 {\textsc {P4P}}\textbf{x} {\textsc {HighSev}} + \beta _7\textbf{X}_i + \epsilon _{ij}. \end{aligned}$$

Following Clark and Huckman (2012), we include the terms $\beta _4 {\textsc {P4P}}\textbf{x} {\textsc {MildSev}}$, $\beta _5 {\textsc {P4P}}\textbf{x} {\textsc {IntermSev}}$, and $\beta _6 {\textsc {P4P}}\textbf{x} {\textsc {HighSev}}$, which interact P4P with each severity-level of illness to determine the extent to which the effect (marginal benefit) of P4P depends on the patient’s severity of illness. By construction, the estimates of $\beta _4$, $\beta _5$, and $\beta _6$ represent the total effect of P4P for patients with either mild, intermediate or high severity of illness, respectively.

Estimation results support Hypothesis 2; see Models (2), (5), and (8). First, P4P positively affects the quantity and quality of care, as all coefficients on the effects are significantly different from zero, except the P4P-effect regarding the quantity for severely-ill patients; see Model (2). Second, we find the hypothesized relation between severity of illness and P4P such that coefficients are significantly higher for less severely-ill patients.

For a patient’s marginal health-benefit, we estimate whether the P4P-effect differs for patients with high and low marginal benefits. We consider a model similar to Equation (2), in which we interact P4P with the marginal health-benefit levels. When comparing the P4P-effect for patients with low marginal health-benefits ($\textsc {P4P}\textbf{x}\textsc {LowMHB}$) to the effect for patients with a high marginal health-benefit ($\textsc {P4P}\textbf{x}\textsc {HighMHB}$), we observe no significant differences; see Models (3), (6), and (9) of Table 2 and the Wald tests. We summarize as follows:

#### Result 2

(**FFS**$+$**P4P and patients’ characteristics**) *While fee-for-service-based performance pay improves the quality of care for all severity types, the performance-pay effect significantly decreases with increasing severity of illness. For patients’ marginal health-benefit, the effect is less systematic.*

### Effects of performance pay blended with capitation

We now analyze how introducing CAP+P4P affects the quantity and quality of care. Brosig-Koch et al. (2024) used the same design to investigate the P4P-effect with general practitioners and medical students when CAP is the baseline payment. We repeat the analyses with our data according to our econometric specifications in Equations (1) and (2). We thus provide the basis for jointly comparing the payment systems FFS, CAP, FFS+P4P, and CAP+P4P in Sect. 3.4.

According to Hypothesis 3, introducing P4P to CAP reduces the underprovision of medical services and enhances the quality of care. Models (1), (4), and (7) of Table 3 show that CAP$+$P4P leads to a highly significant increase in the quantity by on average 1.09 services, a reduction of non-optimal care by on average 1.12 medical services, and an increase in the proportional health-benefit by about 17.5 percentage points. We thus state:

#### Result 3

(**CAP**$+$**P4P**) *Complementing capitation with performance pay leads to a decrease in underprovision and an increase in the quality of care.*

**Table 3:** Regression models on the effect on quantity and quality under CAP conditions

	A. Quantity of medical services *q*			B. Absolute deviation from optimal care $\rho$			C. Proportional health-benefit $\tilde{H}$		
Method:	OLS	OLS	OLS	OLS	OLS	OLS	Frac. Probit	Frac. Probit	Frac. Probit
Model:	(1)	(2)	(3)	(4)	(5)	(6)	(7)	(8)	(9)
P4P	1.085$$^{***}$$			-1.117$$^{***}$$			0.175$$^{***}$$		
	(0.189)			(0.180)			(0.026)		
IntermSev	1.473$$^{***}$$	1.115$$^{***}$$	1.473$$^{***}$$	0.436$$^{***}$$	0.848$$^{***}$$	0.436$$^{***}$$	-0.143$$^{***}$$	-0.201$$^{***}$$	-0.143$$^{***}$$
	(0.100)	(0.139)	(0.100)	(0.074)	(0.138)	(0.074)	(0.016)	(0.024)	(0.016)
HighSev	2.933$$^{***}$$	2.145$$^{***}$$	2.933$$^{***}$$	0.958$$^{***}$$	1.758$$^{***}$$	0.958$$^{***}$$	-0.149$$^{***}$$	-0.227$$^{***}$$	-0.149$$^{***}$$
	(0.151)	(0.245)	(0.151)	(0.134)	(0.250)	(0.134)	(0.019)	(0.028)	(0.019)
HighMHB	0.179$$^{***}$$	0.179$$^{***}$$	0.261$$^{***}$$	-0.115$$^{**}$$	0.115$$^{**}$$	-0.194$$^{**}$$	0.017$$^{**}$$	0.017$$^{**}$$	0.024$$^{***}$$
	(0.048)	(0.048)	(0.069)	(0.044)	(0.044)	(0.073)	(0.007)	(0.007)	(0.009)
P4P$$\times$$ MildSev		0.321$$^{**}$$			-0.309$$^{***}$$			0.055$$^{***}$$	
		(0.140)			(0.108)			(0.017)	
P4P$$\times$$ IntermSev		1.036$$^{***}$$			-1.133$$^{***}$$			0.157$$^{***}$$	
		(0.201)			(0.189)			(0.021)	
P4P$$\times$$ HighSev		1.897$$^{***}$$			-1.909$$^{***}$$			0.180$$^{***}$$	
		(0.296)			(0.292)			(0.023)	
P4P$$\times$$ LowMHB			1.139$$^{***}$$			-1.170$$^{***}$$			0.165$$^{***}$$
			(0.195)			(0.188)			(0.024)
P4P$$\times$$ HighMHB			0.976$$^{***}$$			-1.012$$^{***}$$			0.135$$^{***}$$
			(0.193)			(0.173)			(0.018)
Constant	1.725$$^{***}$$	2.107$$^{***}$$	1.698$$^{***}$$	1.440$$^{***}$$	1.036$$^{***}$$	1.466$$^{***}$$			
	(0.250)	(0.231)	(0.254)	(0.242)	(0.227)	(0.247)			
Wald test (*p*-value)									
$H_0$: P4P$\times$ MildSev$=$ P4P$\times$ IntermSev		<0.001			<0.001			<0.001	
$H_0$: P4P$\times$ MildSev$=$ P4P$\times$ HighSev		<0.001			<0.001			<0.001	
$H_0$: P4P$\times$ IntermSev$=$ P4P$\times$ HighSev		<0.001			<0.001			0.015	
$H_0$: P4P$\times$ LowMHB$=$ P4P$\times$ HighMHB			0.097			0.049			0.004
Observations	990	990	990	990	990	990	990	990	990
Subjects	55	55	55	55	55	55	55	55	55
(Pseudo) $R^{2}$	0.509	0.534	0.509	0.287	0.328	0.287	0.131	0.140	0.131

This table shows parameter estimates from OLS regressions (Panel A and B) and average marginal effects from fractional probit response regressions (Panel C). Robust standard errors clustered for subjects are shown in parentheses. P4P is a dummy variable indicating the introduction of P4P. IntermSev and HighSev are dummy variables for intermediate and high severities of illness. HighMHB is a dummy for the marginal health-benefit being 1 if $\theta = 2$ (high), and 0 otherwise ($\theta = 1$, low). All models control for individual characteristics which comprise gender, medical background (non-medical student, medical student, physician), and personality traits; for the respective estimates, see Table C.6 in Online-Appendix C. * $p<0.10$, ** $p<0.05$, and, *** $p<0.01$

To analyze the effects patients’ characteristics have on the responses of subjects in the role of physicians to CAP+P4P (Hypothesis 4), we again first study the impact the severities of illness have on treatment decisions, as indicated in Equation (1). We find that the quantities for intermediately and severely-ill patients are significantly higher than for mildly-ill patients by, on average, 1.47 and 2.93 medical services, respectively; see Model (1) of Table 3. The quality of care is significantly lower for intermediately-ill (severely-ill) patients deviating by on average 0.44 (0.96) services more from the patient-optimal quantity. The proportional health-benefit is on average 14.3 (14.9) percentage points lower for these patients; see Models (4) and (7) of Table 3.

While the average P4P-effect on the quantity and the quality of care is positive and significant, we find substantial heterogeneity when interacting CAP+P4P with severities: Increases in quantities and the deviation from the patient-optimal quantity ($\rho$) vary significantly for mildly-ill, intermediately-ill, and severely-ill patients. $\tilde{H}$ increases by, on average, 5.5, 15.7, and 18.0 percentage points, respectively; Wald tests indicate significant differences; see Models (2), (5), and (8) of Table 3. The quantity of services for severely-ill patients deviates the most from the patient-optimal quantity, resulting in the lowest proportional health-benefit; see Table C.1 in Online-Appendix C. Severely-ill patients benefit the most from introducing CAP+P4P, which supports Hypothesis 4.

Patients with a high marginal health-benefit receive significantly more medical services and quality of care compared to patients with a low marginal health-benefit. Moreover, while both patient types benefit from CAP$+$P4P, the patients with a low marginal benefit gain more from introducing P4P than those with a high marginal benefit; see Models (6) and (9) of Table 3. This pattern is not in line with Hypothesis 4. However, differences in the P4P-effect for patients with a low and high marginal health-benefit are rather small. Adding interaction terms of marginal health-benefits and P4P does not explain the variation in our data better (comparing Models (1) to (3), (4) to (6), and (7) to (9)). In sum, we state:

#### Result 4

(**CAP**$+$**P4P and patients’ health characteristics**) *The effect of capitation-based performance pay significantly increases in patients’ severities of illness. Patients with a low as well as a high level of marginal health-benefit gain from performance pay; yet, the effect on quality is smaller for patients with a higher marginal benefit.*

Results 3 and 4 are broadly in line with Brosig-Koch et al. (2024). In their study, the P4P-effects for the marginal health-benefit go in the same direction, but they are statistically not significant.

### Comparison of performance-pay effects between blended capitation and fee-for-service systems

While the blended performance-pay systems are symmetric due to their mirror-image design, effect sizes may not be identical, because a decision-maker may perceive the incentives differently. FFS (with fees higher than marginal costs) incentivizes overprovision of care. Under CAP, however, subjects in the role of physicians have an incentive for underprovision as each medical service provided is costly, reduces the lump-sum payment and, therefore, the profit of the respective subjects. Depending on the baseline payment system, introducing P4P provides incentives that go in opposite directions: either to reduce services when complementing FFS or to expand them when blended with CAP.

**Fig. 2:**
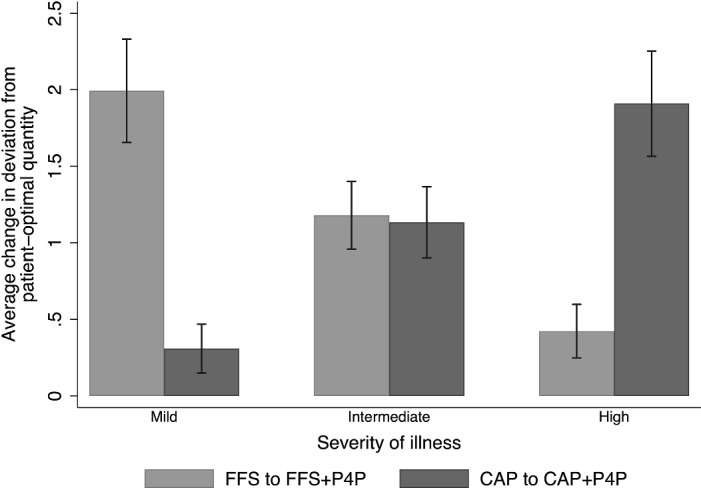
Reduction in the absolute deviation from optimal care by payment system and severity of illness. *Notes.* This figure shows the reduction in $\rho$ achieved by performance pay, differentiated by FFS and CAP conditions and severities of illness

To test Hypothesis 5, we investigate whether the severity-specific effects of P4P on the quality of care differ between FFS+P4P and CAP+P4P. Figure 2 shows that the effects of the blended P4P-systems on $\rho$ strongly vary with the patient’s severity of illness. Table 4 provides descriptive statistics on the two quality measures $\rho$ and $\tilde{H}$ differentiated by patients’ severities of illness and marginal health-benefits. For mildly-ill patients, the improvement in the quality of care is significantly higher under FFS$+$P4P than under CAP$+$P4P ($p<0.001$, two-sided Mann–Whitney U-tests for both quality measures). We observe the reverse pattern for severely-ill patients ($p<0.001$) and no significant differences for patients with an intermediate severity of illness ($p\ge 0.598$). When differentiating by patients’ marginal health-benefit, we observe no significant differences in the P4P-effect across payment conditions ($p\ge 0.308$).

**Table 4:** Descriptives of performance-pay effects

	FFS to FFS$+$P4P	CAP to CAP$+$P4P	Diff	*p*-value
*A. Change in absolute deviation from optimal care *$\rho$				
Aggregate	$-$1.20 (1.73)	$-$1.12 (1.79)	$-$0.08	0.278
Mild severity	$-$1.99 (2.14)	$-$0.31 (1.04)	$-$1.68	<0.001
Intermediate severity	$-$1.18 (1.40)	$-$1.13 (1.51)	$-$0.05	0.598
High severity	$-$0.42 (1.11)	$-$1.91 (2.24)	$-$1.49	<0.001
Low marginal health-benefit	$-$1.21 (1.78)	$-$1.17 (1.87)	$-$0.04	0.519
High marginal health-benefit	$-$1.17 (1.63)	$-$1.01 (1.63)	$-$0.16	0.316
*B. Change in proportional health-benefit * $\tilde{H}$				
Aggregate	0.19 (0.27)	0.18 (0.29)	0.01	0.286
Mild severity	0.28 (0.31)	0.04 (0.15)	0.24	<0.001
Intermediate severity	0.24 (0.28)	0.23 (0.31)	0.01	0.608
High severity	0.06 (0.16)	0.27 (0.32)	0.21	<0.001
Low marginal health-benefit	0.19 (0.28)	0.19 (0.30)	0.00	0.550
High marginal health-benefit	0.19 (0.26)	0.16 (0.26)	0.03	0.308
Observations	468	495		
Subjects	52	55		

The table reports descriptive statistics on the changes in our quality measures $\rho$ and $\tilde{H}$ when moving from unblended to pay-for-performance payment schemes (means; standard deviations in parentheses). We differentiate by patients’ severities of illness and the marginal health-benefit. Column ‘Diff’ reports average differences in effect sizes between both payment schemes; reported *p*-values are based on two-sided Mann–Whitney U tests

To quantify effects, we use regression analyses that extend our basic econometric model by the between-payment system comparison as follows:

3 $$\begin{aligned} y_{ij}= & \alpha + \beta _1 {\textsc {CAP}} + \beta _2 {\textsc {IntermSev}} + \beta _3 {\textsc {HighSev}} + \beta _4 {\textsc {HighMHB}} \nonumber \\ & + \beta _5 {\textsc {CAP}}\times {\textsc {IntermSev}} + \beta _6 {\textsc {CAP}}\times {\textsc {HighSev}} \nonumber \\ & + \beta _7 {\textsc {CAP}+\textsc{P4P}}\times {\textsc {MildSev}} + \beta _8 {\textsc {FFS}+\textsc{P4P}}\times {\textsc {MildSev}}\nonumber \\ & + \beta _9 {\textsc {CAP}+\textsc{P4P}}\times {\textsc {IntermSev}} + \beta _{10} {\textsc {FFS}+\textsc{P4P}}\times {\textsc {IntermSev}} \nonumber \\ & + \beta _{11} {\textsc {CAP}+\textsc{P4P}} \times {\textsc {HighSev}} + \beta _{12} {\textsc {FFS}+\textsc{P4P}} \times {\textsc {HighSev}} \nonumber \\ & + \beta _{13} \textbf{X}_i + \epsilon _{ij}, \end{aligned}$$

The variable CAP is a dummy which equals 1 if a subject in the role of a physician is remunerated by CAP, and 0 if he or she is remunerated by FFS. IntermSev$\times$ CAP and HighSev$\times$ CAP show interaction effects between CAP and the respective level of severity. To determine how severity-specific effects of P4P vary by the underlying remuneration condition, we interact the variables CAP+P4P and FFS+P4P (which are dummies for the respective blended payment systems) with each level of severity. The estimate for $\beta _7$ thus represents the total effect of P4P for mildly-ill patients under CAP, while $\beta _8$ represents the total effect for mildly-ill patients under FFS. $\beta _9$ to $\beta _{12}$ represent the respective effects for the remaining illnesses. $X_{i}$ is the vector of subject *i*’s characteristics comprising gender, medical background and personality traits.

Estimation results and Wald-Tests show that differences in performance-pay effects between FFS$+$P4P and CAP$+$P4P exist and are severity-specific; see Table 5. The result is robust towards individual characteristics (comparing Models (1) to (2) and (3) to (4) of Table 5).

**Table 5:** Comparison of effects of blended performance pay systems

	A. Absolute deviation from patient-optimal care $\rho$			B. Proportional health-benefit $\tilde{H}$	
Method	OLS	OLS		Frac. Probit	Frac. Probit
Model	(1)	(2)		(3)	(4)
CAP	$$-$$1.828$$^{***}$$	$$-$$1.832$$^{***}$$		0.210$$^{***}$$	0.209$$^{***}$$
	(0.342)	(0.310)		(0.037)	(0.032)
IntermSev	$$-$$0.936$$^{***}$$	$$-$$0.936$$^{***}$$		0.020$$^{*}$$	0.020
	(0.147)	(0.148)		(0.012)	(0.012)
HighSev	$$-$$1.782$$^{***}$$	$$-$$1.782$$^{***}$$		0.182$$^{***}$$	0.178$$^{***}$$
	(0.253)	(0.254)		(0.021)	(0.020)
HighMHB	$$-$$0.086$$^{**}$$	$$-$$0.086$$^{**}$$		0.013$$^{**}$$	0.013$$^{**}$$
	(0.033)	(0.034)		(0.005)	(0.005)
CAP$$\times$$ IntermSev	1.784$$^{***}$$	1.784$$^{***}$$		$$-$$0.243$$^{***}$$	$$-$$0.241$$^{***}$$
	(0.201)	(0.201)		(0.029)	(0.028)
CAP$$\times$$ HighSev	3.540$$^{***}$$	3.540$$^{***}$$		$$-$$0.492$$^{***}$$	$$-$$0.484$$^{***}$$
	(0.354)	(0.355)		(0.033)	(0.033)
CAP+P4P$$\times$$ MildSev	$$-$$0.309$$^{***}$$	$$-$$0.309$$^{***}$$		0.056$$^{***}$$	0.054$$^{***}$$
	(0.107)	(0.107)		(0.017)	(0.016)
FFS+P4P$$\times$$ MildSev	$$-$$1.994$$^{***}$$	$$-$$1.994$$^{***}$$		0.171$$^{***}$$	0.170$$^{***}$$
	(0.274)	(0.275)		(0.017)	(0.016)
CAP+P4P$$\times$$ IntermSev	$$-$$1.133$$^{***}$$	$$-$$1.133$$^{***}$$		0.148$$^{***}$$	0.148$$^{***}$$
	(0.187)	(0.188)		(0.018)	(0.017)
FFS+P4P$$\times$$ IntermSev	$$-$$1.179$$^{***}$$	$$-$$1.179$$^{***}$$		0.151$$^{***}$$	0.150$$^{***}$$
	(0.181)	(0.182)		(0.017)	(0.016)
CAP+P4P$$\times$$ HighSev	$$-$$1.909$$^{***}$$	$$-$$1.909$$^{***}$$		0.168$$^{***}$$	0.167$$^{***}$$
	(0.289)	(0.290)		(0.018)	(0.017)
FFS+P4P$$\times$$ HighSev	$$-$$0.423$$^{***}$$	$$-$$0.423$$^{***}$$		0.075$$^{***}$$	0.077$$^{***}$$
	(0.110)	(0.111)		(0.015)	(0.015)
Constant	2.759$$^{***}$$	2.950$$^{***}$$			
	(0.316)	(0.324)			
Individual controls	No	Yes		No	Yes
Wald tests (*p*-value)					
$H_0$: CAP+P4P$\times$ MildSev $=$ FFS+P4P$\times$ MildSev	<0.001	<0.001		<0.001	<0.001
$H_0$: CAP+P4P$\times$ IntermSev $=$ FFS+P4P$\times$ IntermSev	0.860	0.860		0.872	0.884
$H_0$: CAP+P4P$\times$ HighSev $=$ FFS+P4P$\times$ HighSev	<0.001	<0.001		<0.001	<0.001
Observations	1926	1926		1926	1926
Subjects	107	107		107	107
(Pseudo) $R^{2}$	0.240	0.312		0.094	0.129

For Panel A, OLS estimates are reported with robust standard errors clustered for subjects (in brackets). For Panel B, average marginal effects (AMEs), based on a fractional probit response model, are reported with robust standard errors clustered for subjects (in brackets). CAP = 1 if physicians are remunerated by CAP, and = 0 otherwise (by FFS). P4P is a dummy variable indicating the introduction of P4P. IntermSev and HighSev are dummy variables for intermediate and high severities of illness. HighMHB is a dummy for the marginal health-benefit being 1 if $\theta = 2$ (high), and 0 otherwise ($\theta = 1$, low). Controls for subjects’ individual characteristics comprise gender, medical background (non-medical student, medical student, physician), and personality traits; for the respective estimates, see Table C.7 in Online-Appendix C. * $p<0.10$, ** $p<0.05$, and *** $p<0.01$

Our finding that P4P-effects are severity-specific supports Hypothesis 5. We observe that the marginal benefit of P4P on the quality of care is highest for mildly-ill patients under FFS$+$P4P. Models (2) and (4) of Table 5 show that the absolute deviation from the patient-optimal quantity is reduced by on average 1.99 medical services, and the patients’ health-benefit increases by about 17.0 percentage points. On the contrary, the effect is lowest for mildly-ill patients under CAP$+$P4P. Estimates indicate a reduction in $\rho$ by about 0.31 medical services and an increase in $\tilde{H}$ by 5.4 percentage points. The introduction of P4P is therefore 6.5 times (3.1 times) more effective in terms of $\rho$ ($\tilde{H}$) for mildly-ill patients under FFS$+$P4P than under CAP$+$P4P.

For severely-ill patients, the estimates show a reverse pattern in that the P4P-effect is significantly higher under CAP$+$P4P compared to FFS$+$P4P. P4P leads to a reduction in $\rho$ by on average 1.91 medical services under CAP$+$P4P and by 0.42 medical services under FFS$+$P4P. $\tilde{H}$ increases by 16.7 (7.7) percentage points under CAP$+$P4P (FFS$+$P4P).

For intermediately-ill patients, we find no significant difference in P4P-effects between payment systems. Put differently, the introduction of P4P yields similar quality improvements for intermediately-ill patients, which lead to a reduction of about 1.13 (1.18) medical services in $\rho$ and a higher $\tilde{H}$ by about 14.8 (15.0) percentage points under CAP$+$P4P (FFS$+$P4P). In sum, we state the following result:

**Result 5 (Comparisons of FFS+P4P and CAP+P4P).** *The performance-pay effect on the quality of care is specific to the patient’s severity of illness across the two blended pay-for-performance systems. While the effect on the quality of care is significantly higher for mildly-ill patients under FFS*$+$*P4P, it is significantly higher for severely-ill patients under CAP*$+$*P4P. For intermediately-ill patients the effect of performance pay on the quality of care does not differ between payment systems.*

## Discussion

To put behavioral results into context, we now discuss benefits and costs of introducing performance pay. Most research on the effects of initiating a P4P-system focuses on quality measure targets, thereby often neglecting the pertinent issues of individual health outcomes and costs (e.g., Meacock et al., 2014). We address this issue within the confines of our experimental setting.

The average patient health-benefit ($\overline{H}$) is 7.92 in FFS and 8.01 in CAP; see Table 6. $\overline{H}$ significantly increases to 9.47 in FFS$+$P4P and to 9.51 in CAP$+$P4P ($p< 0.001$, Wilcoxon signed rank-test). Also, the remuneration of subjects in the role of physicians increases significantly ($p<0.001$, Wilcoxon signed rank-test). This is in line with earlier studies (e.g., Mullen et al., 2010) and does not come at a surprise, as subjects react to the P4P-incentives in our experiment.

**Table 6:** Patients’ benefits, costs for physicians’ remuneration, and changes in costs and benefits

	Aggregated			Mild severity			Interm. severity			High severity	
	$\overline{H}$	$\overline{R}$		$\overline{H}$	$\overline{R}$		$\overline{H}$	$\overline{R}$		$\overline{H}$	$\overline{R}$
FFS	7.92	13.38		6.72	11.38		7.92	13.38		9.10	15.38
FFS$+$P4P	9.51	15.02		9.35	12.93		9.52	14.75		9.65	17.38
Change	1.59	1.64		2.63	1.55		1.60	1.37		0.55	2.00
Ratio ($\Delta$ $R$/$\Delta$ $H$)	1.03			0.59			0.86			3.64	
CAP	8.01	10.00		9.15	10.00		8.03	10.00		6.86	10.00
CAP$+$P4P	9.47	13.77		9.53	12.31		9.51	13.53		9.36	15.46
Change	1.46	3.77		0.38	2.31		1.48	3.53		2.50	5.46
Ratio ($\Delta$ $R$/$\Delta$ $H$)	2.58			6.08			2.39			2.18	

This table shows the average patients’ health-benefits $\overline{H}$ and remuneration $\overline{R}$ for FFS, CAP, FFS$+$P4P, and CAP$+$P4P, both aggregated and differentiated for severities of illness (mild, intermediate, high). It further shows the marginal payment, marginal patient health-benefit, and the ratio of marginal payment to marginal patient health-benefit, also aggregated and separately for the three severities of illness

As it is important to understand the influence of P4P-systems’ design elements (e.g., Kristensen et al., 2016), we take a closer look at costs and benefits for the different severities of illness. We find that patient health-benefits and physician remuneration significantly increase for all severities ($p<0.010$, Wilcoxon signed rank-test). Under CAP, the increase in health-benefit is highest for the severely-ill patients (43.7%), while under FFS it is highest for mildly-ill patients (39.1%). This implies an increase in remuneration by 54.6% for the severely-ill patients under CAP and by 13.6% for the mildly-ill patients in FFS. Differences in relative changes between payment systems indicate that remuneration costs need to be taken into account when assessing the effectiveness of P4P.

We also find, that the financial resources needed to induce a one-unit increase in health-benefit vary substantially between payment systems. On average, 2.58 monetary units in CAP conditions and 1.03 units in FFS conditions are needed for a one-unit increase in health-benefit. Under CAP, the ratio is lowest for severely-ill patients (2.18), due to the large increase in patient health-benefit. The ratio is highest for mildly-ill patients (6.08), driven by the rather small increase of 4.2% in patient health-benefit. Under FFS, the ratio is 0.59 for patients with a mild severity of illness, while for intermediately-ill patients the ratio is 0.86. This implies an increase in remuneration by less than one monetary unit for a one-unit increase in patient health-benefit. For severely-ill patients, the ratio is 3.64.

We are aware that calculating ratios of marginal payment and marginal patient health-benefit from our experimental data can only serve as a rough benchmark. Our results suggest, however, that incentivizing medical service provision with P4P is advisable for policy-makers, aiming primarily at enhancing the patient health-benefit, regardless of the additional costs generated. Taken at face value, introducing P4P for mildly-ill patients under FFS and for severely-ill patients under CAP would be most effective.

Changing the baseline payment system from CAP to FFS and vice versa could provide an alternative to introducing P4P. The ratio of marginal physician payment to marginal patient health-benefit is 0.58 when switching from FFS to CAP for mildly-ill patients, and 2.40 when moving from CAP to FFS for highly-ill patients. Hence, the effects of interchanging the baseline payment systems are similar to those when introducing P4P. The latter option may be favorable, as it leads to an increase in the patient benefits for all severity types at the aggregate.

## Concluding remarks

The effects of performance pay on physicians’ medical service provision and the quality of care are still not well understood. To contribute in narrowing this gap, we conducted controlled laboratory and artefactual field experiments to analyze the causal effect of pay for performance on medical service provision. At a within-subject level, P4P either complements FFS or CAP - with performance thresholds tied to the patient-optimal treatment and adjusted for the levels of the patients’ severity of illness. Under P4P, subjects increase, on average, the quality of care compared to non-blended payments. We further investigate the positive effect of a threshold-based P4P bonus that is adjusted to the patient’s needs or severity of illness and show that, as expected, the intensity of the P4P effect depends substantially on the severity of the patient’s illness. Although this effect is design-driven and could therefore be regarded as a limitation, we believe that it is a valuable information for policy makers: a patient-adjusted bonus payment, a concept similar to that of diagnosis-related groups in hospital payment systems, can mitigate overprovision of medical services under FFS or overprovision under CAP. At a between-subject level, we analyze further how the severity-specific behavioral responses to P4P differ depending on the baseline payment systems. For intermediately-ill patients, the increase in quality of care is nearly the same under both payment systems when introducing P4P. Mildly-ill patients, however, benefit the most when P4P is complementing FFS, while for highly-ill patients, this is the case when P4P is complementing CAP.

Taking a more general perspective, effective research needs to combine and balance insights from methods being highly valid both from an internal and an external point of view. A controlled lab experiment has high internal validity and serves as a complement rather than a substitute for other research methods with high external validity. It could, for instance, work as a ‘wind tunnel study’, which allows us to test with rather low costs for the behavioral effects of important P4P-design elements prior to implementing these elements in a large-scale randomized controlled trial (RCT), or before introducing policy measures in the field (Galizzi & Wiesen, 2018). Moreover, a combination of theory and experiments by economic engineering has improved the design and functioning of markets and institutions (Falk & Heckman, 2009). Examples in healthcare are the matching of doctors to positions in the medical labor market (Roth, 2002), testing clinical decision support systems (Cox et al., 2016a), and analyzing referral fees (Waibel & Wiesen, 2021).

In our experiment, P4P characterized by a 20%-bonus effectively induced a higher quality of medical service provision. This relatively high bonus level reflected the payment increase in the Quality and Outcomes Framework in the UK (see, e.g., Doran et al., 2006). Moreover, adjusting P4P for the patients’ severity of illness reduced the strong overtreatment of low-severity patients under FFS and the strong undertreatment of high-severity patients under CAP. We designed P4P-bonus payments such that performance thresholds are tied to the patient-optimal care, and we precisely varied bonus sizes to account for severity-specific patient benefits. It might not always be feasible to adequately design such relatively high and patient-adjusted P4P-bonus payments outside the laboratory; yet, a general distinction between patient groups of rather high and low medical needs should be possible. In such cases, patients belonging to the former group should be treated under FFS, while patients with little medical needs should be treated under CAP. This approach would guarantee that harm to patients is kept small, which is caused by deviations from the patient-optimal medical care induced by opposing financial incentives between physician profit and patient benefit. While our study shows the behavioral P4P-effects given this specific design, we believe it is an important avenue for future research to further systematically study the effects of different design elements, such as the size of the incentive, the type of payment (monetary or non-monetary), the type of performance incentive (bonus or fine), the type of performance dimension (outcome, structure or process), the type of performance measure (absolute or relative) as for instance proposed by Ogundeji et al. (2018).

The cost-effectiveness analyses of our data shows that the additional expenditures for bonuses rise disproportionately although introducing P4P does induce increases in the patients’ health-benefit. Given the design of the experiment, our calculations are limited to incentive costs. Yet, other ‘cost categories’ might be affected by introducing P4P-like set up/development costs, running costs, provider costs when participating in the scheme, as well as cost savings (Meacock et al., 2014). The latter category seems likely to apply as P4P induces care with superior health outcomes, which in turn will reduce future healthcare costs.

Finally, our behavioral results also evidence heterogeneity in responses to P4P. This calls for future work to better understand what drives this heterogeneity. What is the role, for example, of individuals’ underlying social preferences, attitudes, and personality traits? These individual characteristics might not only explain healthcare workers’ responses to performance pay (e.g., Donato et al., 2017; Brosig-Koch et al., 2024) but also self-selection into payment systems. Understanding how preferences and attitudes predict sorting (e.g., Ashraf et al., 2020) are therefore of great importance for researchers and policy-makers alike.

## Supplementary Information

Below is the link to the electronic supplementary material.

Supplementary file 1 (pdf 2919 KB)

## Footnotes

1 Patients’ health-benefits are measured in monetary terms. The accumulated benefits are then transferred to a charity that supports surgical treatment of real cataract patients. Similar procedures making patients’ benefits salient have been employed in behavioral experiments in health (Hennig-Schmidt et al., 2011; Hennig-Schmidt & Wiesen, 2014; Kesternich et al., 2015; Godager et al., 2016; Brosig-Koch et al., 2016, 2017a, b; Lagarde & Blaauw, 2017; Di Guida et al., 2019; Martinsson & Persson, 2019; Wang et al., 2020; Waibel & Wiesen, 2021; Ge et al., 2022; Brosig-Koch et al., 2024).

2 The participants hence knew exactly how much in monetary terms they received and how much went to the real patients. Although we thereby abstract from everyday medical decisions by not considering risk or ambiguity about patient-benefit, we consider it as an advantage of a well-controlled experimental design as there is no additional confounder on the decision. Evidence on the effects of risk and ambiguity on physicians’ provision behavior from laboratory experiments by Martinsson and Persson (2019) shows that the strong impact of the payment system on physicians’ medical service provision behavior overall remains unaffected by the introduction of risk and ambiguity in patient health outcomes. However, there is substantial heterogeneity across individuals, particularly under a capitation system: more risk-averse (ambiguity-averse) participants provide treatment closer to patients’ optimal treatment levels following the introduction of risk (ambiguity) in patient health.

3 In particular, Brosig-Koch et al. (2024) asked participants about the motives for their decisions in an open question following the study and report that 103 of the 104 participants were motivated solely by patient benefit or by both benefit and their own profit. None of them referred to the design as being too artificial or simplistic. Only two participants described the experimental decision situation as somewhat theoretical and only a vague reflection of their daily experiences in practice, but acknowledged that the incentives and trade-offs inherent in the decision situation were realistic.

4 There were some minor differences in the experimental procedures. Before the experiments, physicians were briefly introduced to the experimental method, the university involved in running the experiment, and the funding institutions of our research project. After the experiment, physicians were debriefed about our purpose and behavioral experiments.

5 Note that practicing physicians are more patient regarding under FFS compared to students, in that they deviate less from the patient-optimal quantity and provide a significantly higher patient benefit, and we observe no difference between medical and non-medical students (Table C.5 Appendix C). Under CAP, however, we find no differences in the impact of medical experience on behavior (Table C.6). These results are comparable to the ones by Brosig-Koch et al. (2016).
