# Supplementary material for: Physicians’ incentives, patients’ characteristics, and quality of care: a systematic experimental comparison of performance-pay systems
Source: Int J Health Econ Manag. 2025 Mar 19;25(2):217–43. doi: 10.1007/s10754-025-09390-x (PMC12360989; doi:10.1007/s10754-025-09390-x)
Supplement: Supplementary file 1 — (pdf 2919 KB) [file 10754_2025_9390_MOESM1_ESM.pdf]

ONLINE APPENDIX

Physicians' incentives, patients' characteristics, and quality of care: A systematic experimental comparison of performance-pay systems*

International Journal of Health Economics and Management

https://doi.org/10.1007/s10754-025-09390-x

Jeannette Brosig-Koch, Mona Groß, Heike Hennig-Schmidt,

Nadja Kairies-Schwarz, Daniel Wiesen

*Brosig-Koch: Otto von Guericke University Magdeburg, Faculty of Economics and Management, and University of Duisburg-Essen, Health Economics Research Center (CINCH) Essen, Germany, e-mail: jeannette.brosig-koch@ovgu.de; Groß: University of Cologne, Department of Health Care Management, Germany; Hennig-Schmidt: University of Bonn, Department of Economics, Germany, e-mail: hschmidt@uni-bonn.de; Kairies-Schwarz: Heinrich Heine University Duesseldorf, Institute for Health Services Research and Health Economics, and German Diabetes Center Duesseldorf, Germany, e-mail: Nadja.Kairies-Schwarz@uni-duesseldorf.de (corresponding author); Wiesen: University of Cologne, Department of Health Care Management, Germany and Erasmus University Rotterdam, Erasmus School of Health Policy & Management (ESHPM), The Netherlands, e-mail: wiesen@wiso.uni-koeln.de.

A Further details about the experiment

A.1 Experimental procedure

The experimental procedure was as follows: Upon arrival, subjects were randomly assigned to workstations separated by panels to ensure that decisions could be made in full anonymity. They then were given ample time to read the instructions for part I. Subjects were informed that the experiment consisted of two parts, but received detailed instructions for part II only after having finished part I of the experiment. For instructions, see Online-Appendix A.3. To check for the subjects' understanding of the decision task, they had to answer a set of control questions. The experiment did not start unless all subjects had answered the control questions correctly. In each of the two parts of the experiment, subjects then subsequently decided on the quantity of medical services for each of the nine patients, i.e., for each possible combination of illnesses and severities. The order of patients was randomly determined and kept constant for all subjects and all conditions: $Bx, Cx, Az, By, Bz, Ay, Cz, Ax, Cy$.

Before making their decision for a specific patient, subjects were informed about their payment, their cost and profit, as well as about the patient benefit for each quantity from 0 to 10. All monetary amounts are given in Taler, our experimental currency. The exchange rate is 1 Taler = EUR 0.80 in the lab experiment and 1 Taler = EUR 3.40 in the artefactual field experiment. Compared to the lab, the payment in the field experiment was increased by a factor of 4.25 to provide adequate incentives for the physicians.[1] The procedure was exactly the same in part II of the experiment. After finishing part II, we asked the subjects to complete a questionnaire on social demographics (e.g., age and gender) and on personality traits elicited by a ten-item personality inventory, which comprises five personality dimensions: extraversion, agreeableness, conscientiousness, neuroticism, and openness (Rammstedt and John, 2007). An overview on summary statistics on social demographics and personality traits can be found in Table A.1 in Appendix A.

At the end of the experiment, when all subjects had made their decisions, we randomly determined one decision in each part of the experiment to be relevant for a subject's actual payoff and the patient benefit. This was done to rule out income effects. Subjects were paid in private according to these two randomly determined decisions.

To verify that the money corresponding to the sum of patient benefits in a session was actually transferred to the charity, we applied a procedure similar to Hennig-Schmidt et al. (2011) and Brosig-Koch et al. (2016, 2017). One of the participants was randomly chosen to be the monitor. After the experiment, the monitor verified that a payment order on the aggregated benefit in the respective session was written to the financial department of the University of Duisburg-Essen to transfer the money to the Christoffel Blindenmission, which used the monetary transfers exclusively to support surgical treatments of cataract patients in a hospital in Masvingo (Zimbabwe) staffed by ophthalmologists from the charity. To avoid motives like compassion for people in developing countries, we did not inform the subjects that the money was assigned to a developing country. The order was sealed in an envelope and the monitor and experimenter then walked together to the nearest mailbox and deposited the envelope. The monitor was paid an additional 5 EUR.

[1]The amount physicians could earn in the experiment was set such that it reflects the average net hourly wage of a physician in Germany, bearing in mind potential differences, for example across the physicians' specialization and seniority. We set this factor after consultation with Dr. Harald Herholz of the Association of Statutory Health Insurance Physicians in Hesse (Germany), who has been involved in budget negotiations for physicians' remuneration.

Laboratory sessions lasted for about 60 minutes. Subjects earned, on average, EUR 16.37. The average benefit per patient was EUR 13.25. In total, EUR 1,152.80 were transferred to the Christoffel Blindenmission. The average cost for a cataract operation amounts, according to the Christoffel Blindenmission, to about EUR 30. Thus, our experiment allowed 38 cataract patients to be treated. The sessions of the artefactual field experiment lasted for about 50 minutes. Physicians earned, on average, EUR 62.73. The average benefit per patient was EUR 67.83. In total, EUR 1,356.60 were transferred to the Christoffel Blindenmission, allowing the treatment of 45 cataract patients.

For a picture of the setup of the mobile lab in Bad Nauheim and the typical setup of the computer laboratory at elfe, see Figure A.1.

Figure A.1: Mobile and computer laboratory

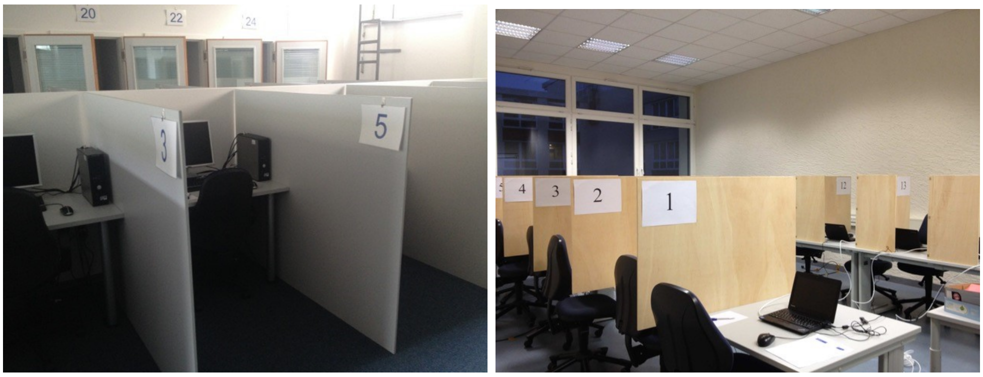

Notes: This figure shows the laboratory setup for the computer laboratory experiments at elfe at the University of Duisburg-Essen (left panel) for our student sample and the mobile laboratory setup of elfe at the Academy for Training and Education in Bad Nauheim for our physician sample.

A.1.1 Sample

Table A.1: Sample characteristics

	Sample			
	Full	Med. students	Non-med. students	Physicians
**A. All payment systems**				
Main characteristics				
Male	40.2%	27.3%	53.5%	40.0%
Age (Mean, s.d.)	28.6 (9.8)	25.2 (7.0)	24.3 (3.4)	45.3 (6.7)
Personality traits (Mean, s.d.)				
Extraversion	3.6 (0.83)	3.7 (0.84)	3.5 (0.90)	3.5 (0.56)
Neuroticism	2.8 (0.97)	2.6 (0.91)	3.0 (0.92)	2.6 (1.10)
Openness	3.6 (0.92)	3.8 (0.88)	3.3 (0.97)	3.6 (0.82)
Conscientiousness	3.6 (0.81)	3.6 (0.69)	3.2 (0.81)	4.3 (0.53)
Agreeableness	3.1 (0.71)	3.3 (0.63)	2.8 (0.73)	3.1 (0.64)
N	107	44	43	20
**B. FFS**				
Main characteristics				
Male	44.2%	40.9%	50.0%	40.0%
Age (Mean, s.d.)	28.4 (9.9)	24.3 (4.5)	24.1 (4.0)	45.9 (7.2)
Personality traits (Mean, s.d.)				
Extraversion	3.7 (0.86)	3.8 (0.89)	3.7 (0.92)	3.4 (0.66)
Neuroticism	2.8 (0.92)	2.4 (0.82)	3.1 (0.85)	3.0 (0.08)
Openness	3.5 (0.93)	3.9 (0.85)	3.3 (1.00)	3.5 (0.80)
Conscientiousness	3.5 (0.79)	3.5 (0.66)	3.1 (0.82)	4.1 (0.61)
Agreeableness	3.1 (0.67)	3.3 (0.66)	2.9 (0.61)	3.2 (0.63)
N	52	22	20	10
**C. CAP**				
Main characteristics				
Male	36.4%	13.6%	56.5%	40.0%
Age (Mean, s.d.)	28.8 (9.9)	26.1 (8.8)	24.6 (2.9)	44.6 (8.2)
Personality traits (Mean, s.d.)				
Extraversion	3.4 (0.79)	3.5 (0.79)	3.4 (0.88)	3.4 (0.58)
Neuroticism	2.7 (1.02)	2.8 (0.99)	2.9 (0.99)	2.3 (1.11)
Openness	3.6 (0.91)	3.7 (0.92)	3.4 (0.95)	4.0 (0.69)
Conscientiousness	3.7 (0.82)	3.8 (0.70)	3.3 (0.81)	4.5 (0.44)
Agreeableness	3.0 (0.76)	3.2 (0.61)	2.9 (0.83)	3.0 (0.90)
N	55	22	23	10

Notes. This table presents summary statistics of subjects' characteristics for (i) the full sample of our experiment, for (ii) medical and (iii) non-medical students in the laboratory experiment and for (iv) physicians in the artifactual field experiment. We further differentiate between payment systems.

A.2 Parameters of the experiment

Figure A.2: Patient health-benefits by illness and severity of illness

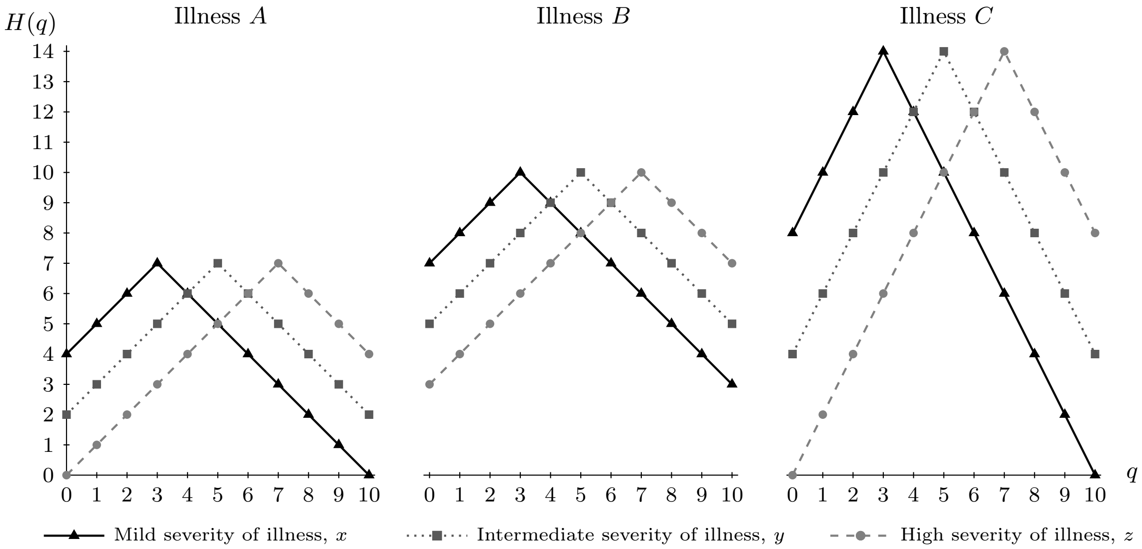

Notes: This figure illustrates patient health-benefit parameters $H(q)$ for illnesses $k = A, B, C$ and severities of illness $l = x, y, z$ on the quantity interval from 0 to 10. The left panel shows patient benefits for illness A, the middle panel for illness B, and the right panel for illness C. The black solid line indicates severity of illness x, the grey dotted line severity of illness y, and the grey dashed line severity of illness z. For illness A and B, $\theta = 1$ and for illness C, $\theta = 2$. Notice that the patient health-benefits are kept constant for all payment conditions.

Figure A.3: Profit parameters in FFS/FFS+P4P and CAP/CAP+P4P

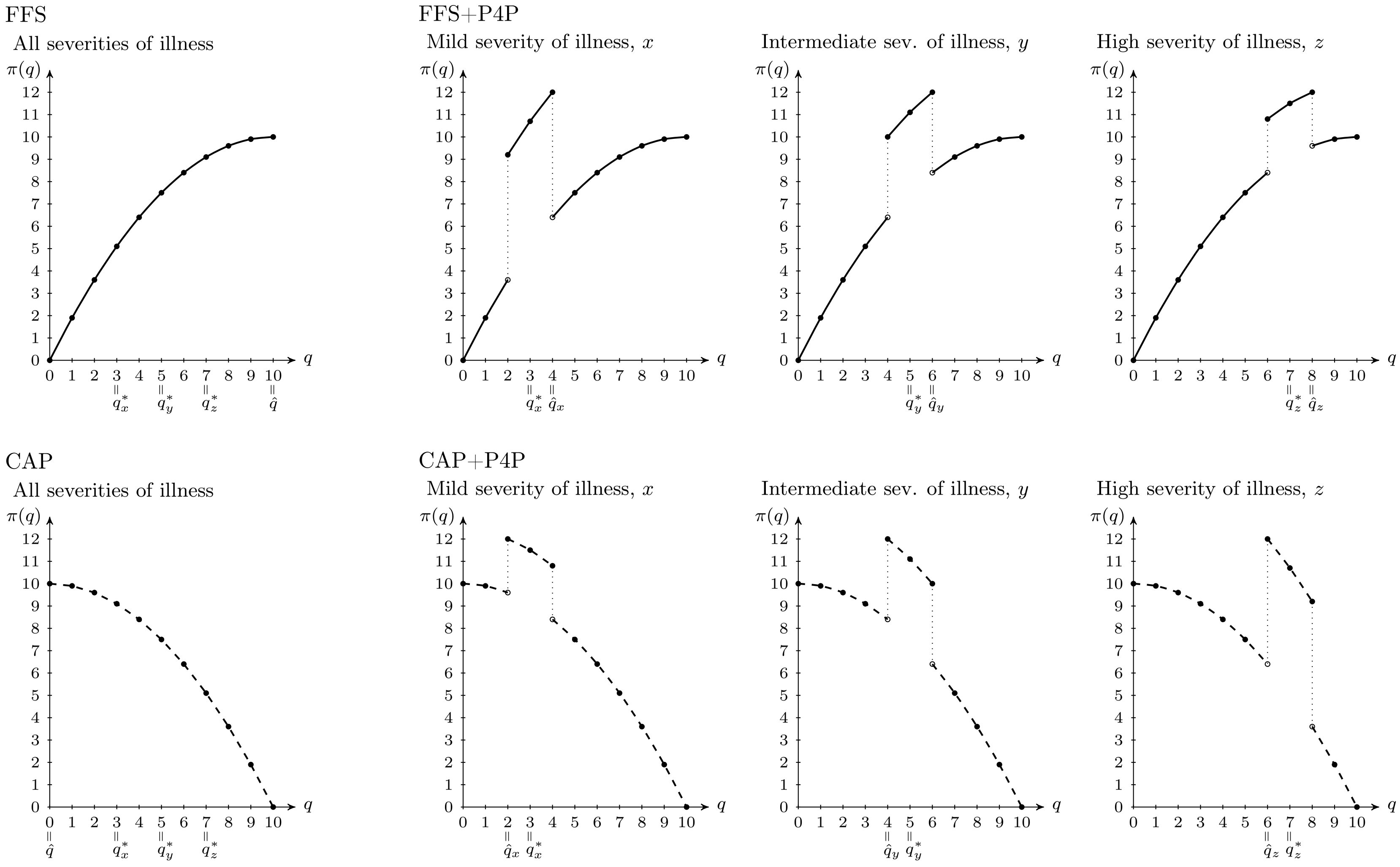

Notes: The upper panel of the figure illustrates profits in FFS and FFS+P4P, the lower panel analogously illustrates profits in CAP and CAP+P4P. Under basic payments, profits increase (in FFS) and decrease (in CAP) continuously, regardless of the severity of illness on the quantity interval. In the pay for performance conditions, a bonus payment is granted if the performance threshold $|q - q^*| \leq 1$ is reached. As the patient-optimal quantity q^* depends on the severity of illness, the performance thresholds differ accordingly. In the basic payment condition, the profit-maximizing quantity is $\hat{q}$=10 in FFS and $\hat{q}$=0 in CAP, respectively. In the pay for performance condition, $\hat{q}$ changes depending on the severity of illness.

Table A.2: Parameters of main experimental conditions

	Quantity (q)										
	0	1	2	3	4	5	6	7	8	9	10
Patient benefit											
B_{Ax}	4	5	6	7	6	5	4	3	2	1	0
B_{Ay}	2	3	4	5	6	7	6	5	4	3	2
B_{Az}	0	1	2	3	4	5	6	7	6	5	4
B_{Bx}	7	8	9	10	9	8	7	6	5	4	3
B_{By}	5	6	7	8	9	10	9	8	7	6	5
B_{Bz}	3	4	5	6	7	8	9	10	9	8	7
B_{Cx}	8	10	12	14	12	10	8	6	4	2	0
B_{Cy}	4	6	8	10	12	14	12	10	8	6	4
B_{Cz}	0	2	4	6	8	10	12	14	12	10	8
Costs											
c	0.0	0.1	0.4	0.9	1.6	2.5	3.6	4.9	6.4	8.1	10.0
FFS											
p	0.0	2.0	4.0	6.0	8.0	10.0	12.0	14.0	16.0	18.0	20.0
π	0.0	1.9	3.6	5.1	6.4	7.5	8.4	9.1	9.6	9.9	10.0
CAP											
L	10.0	10.0	10.0	10.0	10.0	10.0	10.0	10.0	10.0	10.0	10.0
π	10.0	9.9	9.6	9.1	8.4	7.5	6.4	5.1	3.6	1.9	0.0
FFS+P4P											
p	0.0	2.0	4.0	6.0	8.0	10.0	12.0	14.0	16.0	18.0	20.0
b_x	0.0	0.0	5.6	5.6	5.6	0.0	0.0	0.0	0.0	0.0	0.0
b_y	0.0	0.0	0.0	0.0	3.6	3.6	3.6	0.0	0.0	0.0	0.0
b_z	0.0	0.0	0.0	0.0	0.0	0.0	2.4	2.4	2.4	0.0	0.0
π_x	0.0	1.9	9.2	10.7	12.0	7.5	8.4	9.1	9.6	9.9	10.0
π_y	0.0	1.9	3.6	5.1	10.0	11.1	12.0	9.1	9.6	9.9	10.0
π_z	0.0	1.9	3.6	5.1	6.4	7.5	10.8	11.5	12.0	9.9	10.0
CAP+P4P											
L	10.0	10.0	10.0	10.0	10.0	10.0	10.0	10.0	10.0	10.0	10.0
b_x	0.0	0.0	2.4	2.4	2.4	0.0	0.0	0.0	0.0	0.0	0.0
b_y	0.0	0.0	0.0	0.0	3.6	3.6	3.6	0.0	0.0	0.0	0.0
b_z	0.0	0.0	0.0	0.0	0.0	0.0	5.6	5.6	5.6	0.0	0.0
π_x	10.0	9.9	12.0	11.5	10.8	7.5	6.4	5.1	3.6	1.9	0.0
π_y	10.0	9.9	9.6	9.1	12.0	11.1	10.0	5.1	3.6	1.9	0.0
π_z	10.0	9.9	9.6	9.1	8.4	7.5	12.0	10.7	9.2	1.9	0.0

Notes: This table shows the parameters used in our experiment for all payment conditions. p is the fee per service rendered to a patient in FFS, L is the lump-sum payment in CAP, $b_l^\bullet$ is the bonus paid when the quality requirement is met in FFS+P4P (CAP+P4P), and π is the physician's profit.

A.3 Instructions of the experiment

Notice that the text in squared brackets denotes [Capitation, CAP] conditions.

Welcome to the Experiment!

You are participating in an economic experiment on decision behavior. You and the other participants will be asked to make decisions for which you can earn money. Your payoff depends on the decisions you make. At the end of the experiment, your payoff will be converted to Euro and paid to you in cash. During the experiment, all amounts are presented in the experimental currency Taler. 10 Taler equal 8 Euro. The experiment will take about 90 minutes and consists of two parts. You will receive detailed instructions before each part. Note that none of your decisions in either part have any influence on the other part of the experiment.

Part I of the experiment

Please read the instructions carefully. We will approach you in about five minutes to answer any questions you may have. If you have questions at any time during the experiment, please raise your hand and we will come to you. Part I of the experiment consists of 9 rounds of decision situations.

Decision situation

In each round, you are in the role of a physician and decide on medical treatment for a patient. That is, you determine the quantity of medical services you wish to provide to the patient for a given illness and a given severity of this illness. Each patient is characterized by one of three illnesses (A, B, C), each of which can occur in three different degrees of severity (x, y, z). In each consecutive decision round, you will face one patient who is characterized by one of the 9 possible combinations of illnesses and degrees of severity (in random order). Your decision is to provide each of these 9 patients with a quantity of $0, 1, 2, 3, 4, 5, 6, 7, 8, 9$, or 10 medical services.

Payment

In each round, you receive a fee-for-service [capitation] remuneration for treating the patient. Your remuneration increases with the amount of medical treatment [is irrespective of the amount of medical treatment] you provide. You also incur costs for treating the patient, which likewise depend on the quantity of services you provide. Your profit for each decision is calculated by subtracting these costs from the fee-for-service [capitation] remuneration. Each quantity of medical service yields a particular benefit for the patient—contingent on his illness and severity. Hence, in choosing the medical services you provide, you determine not only your own profit but also the patient's benefit.

In each round you will receive detailed information on your screen (see below) for the respective patient, the illness, your amount of fee-for-service [capitation] remuneration—for each possible amount of medical treatment—your costs, profit, as well as the benefit for the patient with the corresponding illness and severity.

Fee-for-service, FFS:

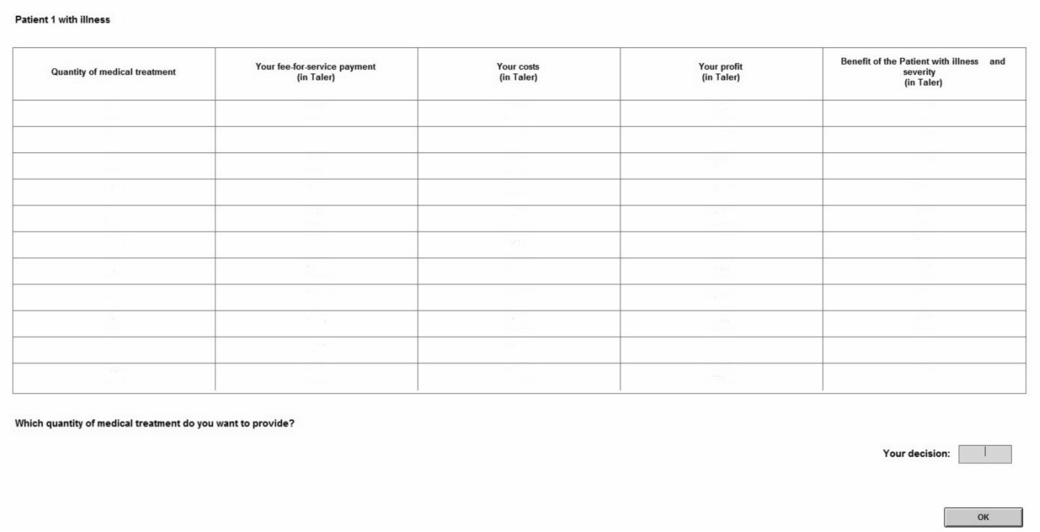

Patient 1 with illness

Quantity of medical treatment	Your fee-for-service payment (in Taler)	Your costs (in Taler)	Your profit (in Taler)	Benefit of the Patient with illness and severity (in Taler)

Which quantity of medical treatment do you want to provide?

Your decision:

OK

[Capitation, CAP:]

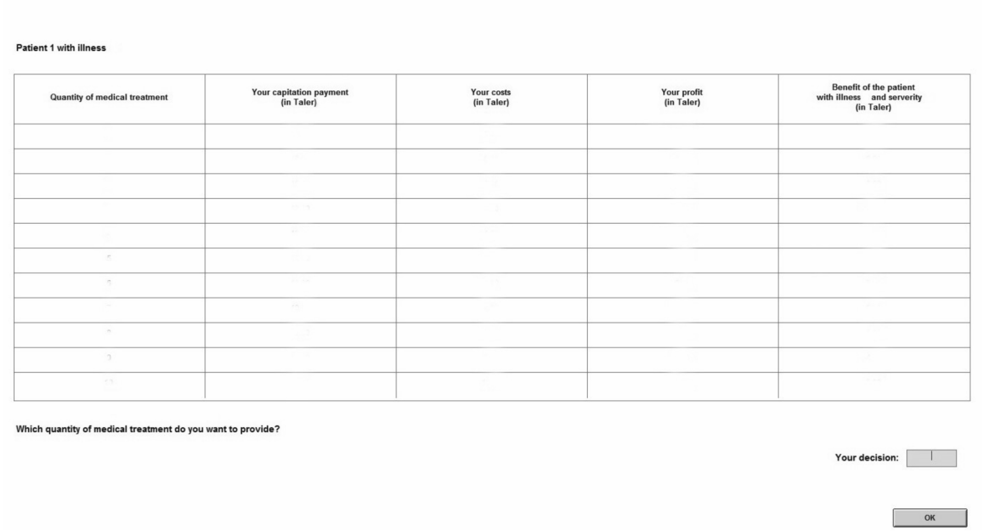

Patient 1 with illness

Quantity of medical treatment	Your capitation payment (in Taler)	Your costs (in Taler)	Your profit (in Taler)	Benefit of the patient with illness and serverity (in Taler)

Which quantity of medical treatment do you want to provide?

Your decision:

OK

Payoff

At the end of the experiment, one of the 9 rounds in part *I* will be chosen at random. Your profit in that round will be paid to you in cash.

For this part of the experiment, no patients are physically present in the laboratory. Yet the patient benefit does accrue to a real patient: The amount resulting from your decision will be transferred to the Christoffel Blindenmission Deutschland e.V., 64625 Bensheim, which will use the money for enabling the treatment of patients with eye cataract.

The transfer of money to the Christoffel Blindenmission Deutschland e.V. will be carried out after the experiment by the experimenter and one participant. The participant completes a money transfer form, filling in the total patient benefit (in Euro) resulting from the decisions made by all participants in the randomly chosen situation. This form prompts the payment of the designated amount to the Christoffel Blindenmission Deutschland e.V. by the finance department of the University of Duisburg-Essen. The

form is then sealed in a stamped envelope and deposited in the nearest mailbox by the participant and the experimenter.

After the entire experiment is completed, one participant is chosen at random to oversee the money transfer to the Christoffel Blindenmission Deutschland e.V. The participant receives an additional compensation of 5 Euro for this task. The participant certifies that the process has been completed as described here by signing a statement that can be inspected by all participants at the office of the Chair of Quantitative Economic Policy. A receipt of the bank transfer to the Christoffel Blindenmission Deutschland e.V. may also be viewed here.

Comprehension questions

Prior to the decision rounds, we kindly ask you to answer a few comprehension questions. They are intended to help you familiarize yourself with the decision situations. If you have any questions about this, please raise your hand. Part I of the experiment will begin once all participants have answered all comprehension questions correctly.

Part II of the experiment

Please read the instructions carefully. We will approach you in about five minutes to answer any questions you may have. If you have questions at any time during the experiment, please raise your hand and we will come to you. Part II of the experiment also consists of 9 rounds of decision situations.

Decision situation

As in part I of the experiment, you take on the role of a physician in each round and decide on medical treatment for a patient. That is, you determine the quantity of medical services you wish to provide to the patient for a given illness and a given severity of this illness.

Each patient is characterized by one of three illnesses (A, B, C), each of which can occur in three different degrees of severity (x, y, z). In each consecutive decision round, you will face one patient who is characterized by one of the 9 possible combinations of illnesses and degrees of severity (in random order). Your decision is to provide each of these 9 patients with a quantity of $0, 1, 2, 3, 4, 5, 6, 7, 8, 9$, or 10 medical services.

Payment

In each round, you are remunerated for treating the patient. In each round, you receive a fee-for-service [capitation] remuneration for treating the patient. Your remuneration increases with the amount of medical treatment [is irrespective of the amount of medical treatment] you provide. In addition to this, in each round you receive a bonus payment, in case the quantity of medical services you provide is equal to the one that results in the highest benefit for the patient, or deviates by one quantity from the latter. You also incur costs for treating the patient, which likewise depend on the quantity of services you provide. Your profit for each decision is calculated by subtracting these costs from the sum of your fee-for-service [capitation] remuneration and bonus payment.

As in part I, every quantity of medical service yields a particular benefit for the patient contingent on his illness and severity. Hence, in choosing the medical services you provide, you determine not only your own profit, but also the patient's benefit.

In each round, you will receive detailed information on your screen (see below) for the respective patient, the illness, your amount of fee-for-service [capitation] remuneration—for each possible amount of medical treatment, the amount of your bonus payment, your costs, profit, as well as the benefit for the patient with the corresponding illness and severity.

FFS+P4P:

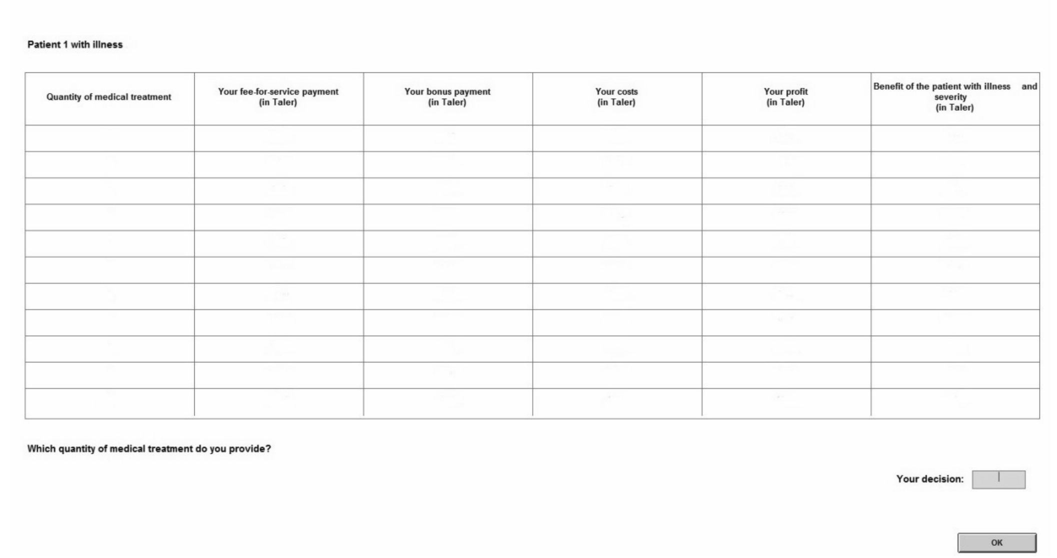

Patient 1 with illness

Quantity of medical treatment	Your fee-for-service payment (in Taler)	Your bonus payment (in Taler)	Your costs (in Taler)	Your profit (in Taler)	Benefit of the patient with illness and severity (in Taler)

Which quantity of medical treatment do you provide?

Your decision:

OK

[CAP+P4P:]

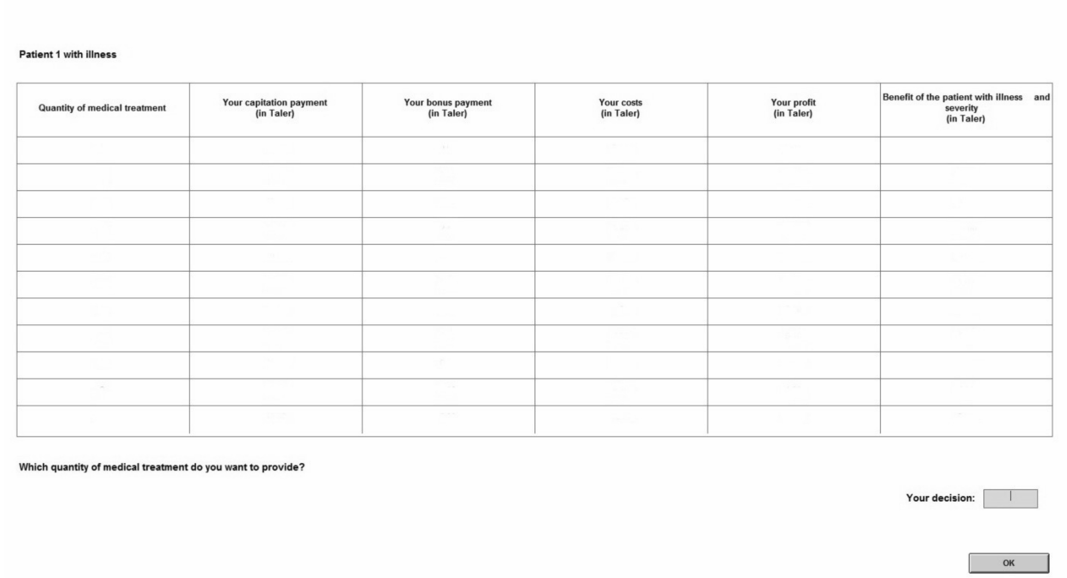

Patient 1 with illness

Quantity of medical treatment	Your capitation payment (in Taler)	Your bonus payment (in Taler)	Your costs (in Taler)	Your profit (in Taler)	Benefit of the patient with illness and severity (in Taler)

Which quantity of medical treatment do you want to provide?

Your decision:

OK

Payoff

At the end of the experiment, one of the 9 rounds of part II will be chosen at random. Your profit in this round will be paid to you in cash, in addition to your payment from the round chosen for part I of the experiment. After the experiment is over, please remain seated until the experimenter asks you to

step forward. You will receive your payment at the front of the laboratory before exiting the room.

As in part I, no patients are physically present in the laboratory for part II of the experiment. Yet the patient benefit does accrue to a real patient: The amount resulting from your decision will be transferred to the Christoffel Blindenmission Deutschland e.V., 64625 Bensheim, which will use the money for enabling the treatment of patients with eye cataract.

The process for transferring the money to the Christoffel Blindenmission Deutschland e.V., as described for part I of the experiment, will be carried out by the experimenter and one participant.

Comprehension Questions

Prior to the decision rounds, we kindly ask you to answer a few comprehension questions. They are intended to help you familiarize yourself with the decision situations. If you have any questions about this, please raise your hand. Part II of the experiment will begin once all participants have answered all comprehension questions correctly.

Finally, we kindly ask you to not talk to anyone about the content of this session in order to prevent influencing other participants after you. Thank you for your cooperation!

B Behavioral predictions

Let physician i choose the quantity of medical services q in order to maximize her utility

$$U_i(q) = \alpha_i H(q) + (1 - \alpha_i)\pi(q), \tag{1}$$

with $\alpha_i \in [0, 1)$. α_i is a measure for physician i's altruism. For a purely profit-maximizing physician, for example, $\alpha_i = 0$. A profit-maximizing physician therefore obtains the highest utility, in the absence of P4P in our experiment, when choosing 10 medical services in FFS and when choosing 0 medical services in CAP.

First, we consider physician i's behavior under the baseline payment systems, i.e., FFS and CAP. For profits and patient benefits given in our experiment, and the given altruism of physician i, we state the following lemma:[2]

Lemma 1. *Physician i overprovides medical services ($q > q^*$) if $p > q^*/5 + (\alpha_i/(1 - \alpha_i))\,\theta$, and she underprovides medical services ($q < q^*$) if $p < q^*/5 - (\alpha_i/(1 - \alpha_i))\,\theta$. Otherwise, physician i chooses the patient optimal quantity ($q = q^*$).*

Proof. Physician i's objective function $U_i(q) = \alpha_i H(q) + (1 - \alpha_i)\pi(q)$ is concave. Payment $R(q) = L + pq$ is linear and $-c(q)$ is concave as $c(q)$ is convex, thus $\pi(q)$ is a concave function. As $H(q)$ is also a concave function and $\alpha_i \geq 0$, it follows that $U_i(q)$ is concave.

Note that as $H(q)$ is not differentiable at $q = q^*$, with $q^* \in (0, 10)$. For $q < q^*$, the first-order condition $U_i'(q) = (1 - \alpha_i)\left[p - \frac{q}{5}\right] + \alpha_i\theta$. For $q > q^*$, the first-order condition $U'(q) = (1 - \alpha_i)_i\left[p - \frac{q}{5}\right] - \alpha_i\theta$. For $q > q^*$, consider $\lim_{q \to q^*} U_i'(q) = (1 - \alpha_i)_i\left[p - \frac{q^*}{5}\right] - \alpha_i\theta$. If $p < q^*/5 - (\alpha_i/(1 - \alpha_i))\,\theta$, $\lim_{q \to q^*} U_i'(q)$ is positive. Also, because $U_i(q)$ is concave, $U_i'(q) > 0\ \forall\ q < q^*$. Therefore any q such that $q \leq q^*$ cannot be optimal, i.e., physician i chooses $q > q^*$.

Analogously for $q < q^*$, consider $\lim_{q \to q^*} U_i'(q) = (1 - \alpha_i)_i\left[p - \frac{q^*}{5}\right] + \alpha_i\theta$. If $p < q^*/5 + (\alpha_i/(1 - \alpha_i))\,\theta$, $\lim_{q \to q^*} U_i'(q)$ is negative. Also because $U_i(q)$ is concave, $U_i'(q) < 0\ \forall\ q > q^*$. Therefore any q such that $q \geq q^*$ cannot be optimal, i.e., physician i chooses $q < q^*$. □

It directly follows from Lemma 1 that physician i's provision behavior depends on the severity of illness (i.e., the patient-optimal quantity q^* varying with severity of illness l), the fee for a medical service p, the marginal patient health-benefit θ, and α_i, the physician i's degree of altruism. Intuitively, the higher physician i's altruism is towards her patient, the lower the degree of non-optimal service provision is. Based on Lemma 1, we expect that FFS induces overprovision of medical services, which decreases in the severity of a patient's illness and in patients' marginal health-benefit. On the contrary, we expect that CAP induces underprovision of medical services, which increases in the severity of a patient's illness, it decreases in patients' marginal health-benefit.

We now focus on the effect of introducing P4P on physicians' healthcare service provision. Comparing physician i's provision behavior between FFS (CAP) and FFS+P4P (CAP+P4P), we state the following proposition:

[2]Notice that Lemma 1 is a special case of Proposition 1 in Brosig-Koch et al. (2017). They consider a physician's behavior under mixed payment systems with a weight on a FFS component and a lump-sum CAP.

Proposition 1. *Performance pay linked to the optimal patient's health-benefit reduces physicians' overprovision of medical services in fee-for-service and underprovision in capitation.*

Proof. Let $q^{\text{Opt.}}$ be a physician's utility-maximizing choice for a patient j under FFS or CAP. Depending on a physician's quantity choice, we distinguish three cases. First, we consider $q^{\text{Opt.}} \in [q^* - 1, q^* + 1]$. As $b_l > 0$, it follows that a physician with $\alpha_i \in [0, 1)$ does not change her behavior since $b_l > 0$ is a constant. Second, we consider $q^{\text{Opt.}} > q^* + 1$. Here, the physician chooses q according to $\max\{U^{II}(q^{\text{Opt.}}), U(q^* + 1) + b_l\}$. That means a physician either does not change her behavior or chooses $q^* + 1$ when P4P has been introduced. Analogously for $q^{\text{Opt.}} < q^* - 1$, the same logic applies. □

Intuitively, whether a physician meets the quality threshold ($|q - q^*| \leq 1$) depends on physician i's degree of altruism towards the patient, according to Lemma 1, counterbalancing the incentive effects in FFS and CAP. For a physician's given altruism with $\alpha_i \in [0, 1)$, introducing P4P, therefore, reduces non-optimal service provision under FFS and CAP. Since former experimental evidence shows that non-optimal service provision is highest for those patients for whom the difference between the incentive effects in FFS and CAP and the patient's optimal quantity are the most misaligned, $\hat{q}$ i.e., for mild severe ill patients under FFS and high severe ill patients under CAP, the effect sizes of P4P are also likely to vary between severity types. Thus, for a physician's given altruism with $\alpha_i \in [0, 1)$, we expect a larger effect of P4P on non-optimal service provision with increasing severity of illness under CAP and decreasing severity under FFS.

C Additional analyses

Table C.1: Quantities and qualities of medical service provision by patients' health characteristics and payment schemes

	FFS		FFS+P4P			CAP		CAP+P4P		
	Mean	s.d.	Mean	s.d.	p-value	Mean	s.d.	Mean	s.d.	p-value
**A. Quantity of medical services** q										
Aggregate	6.69	2.07	5.59	1.66	<0.001	3.32	2.13	4.40	1.71	<0.001
Mild severity	5.69	2.44	3.70	0.64	<0.001	2.23	1.22	2.55	0.83	0.0095
Intermediate severity	6.69	1.74	5.58	0.53	<0.001	3.35	1.84	4.38	0.72	<0.001
High severity	7.69	1.36	7.50	0.56	0.0759	4.38	2.55	6.27	0.81	<0.001
Low marginal health-benefit	6.69	2.12	5.61	1.67	<0.001	3.23	2.18	4.37	1.69	<0.001
High marginal health-benefit	6.70	1.96	5.56	1.63	<0.001	3.49	2.03	4.47	1.74	<0.001
**B. Absolute deviation from patient-optimal care** ρ										
Aggregate	1.82	1.95	0.63	0.55	<0.001	1.77	2.01	0.65	0.75	<0.001
Mild severity	2.73	2.40	0.74	0.59	<0.001	0.90	1.12	0.59	0.73	0.0035
Intermediate severity	1.79	1.64	0.62	0.49	<0.001	1.75	1.75	0.62	0.72	<0.001
High severity	0.95	1.19	0.53	0.54	<0.001	2.66	2.51	0.75	0.78	<0.001
Low marginal health-benefit	1.85	2.00	0.64	0.54	<0.001	1.84	2.08	0.67	0.74	<0.001
High marginal health-benefit	1.76	1.85	0.60	0.56	<0.001	1.64	1.86	0.63	0.77	<0.001
**C. Proportional health-benefit** $\hat{H}$										
Aggregate	0.71	0.31	0.90	0.09	<0.001	0.71	0.32	0.90	0.12	<0.001
Mild severity	0.61	0.34	0.89	0.08	<0.001	0.87	0.16	0.92	0.10	0.0039
Intermediate severity	0.64	0.33	0.88	0.1	<0.001	0.65	0.35	0.88	0.14	<0.001
High severity	0.86	0.17	0.92	0.08	<0.001	0.62	0.36	0.89	0.11	<0.001
High marginal health-benefit	0.71	0.30	0.90	0.09	<0.001	0.73	0.30	0.90	0.13	<0.001
Observations	468		468			495		495		
Subjects	52		52			55		55		

Notes: This table shows descriptive statistics on the quantity and quality of medical service provision for each payment condition, at the aggregate level and differentiated by patients' characteristics (levels of severity of illness and marginal health-benefit). Two-sided p-values are shown for Wilcoxon sigend rank tests for differences in the quantity and quality measures across non-blended (FFS or CAP) and blended payment system (FFS+P4P or CAP+P4P, respectively).

Table C.2: Quantity and quality of healthcare provision by payment system, illness, and severity of illness

	**A. Quantity of medical services** q				**B. Absolute deviation from optimal care** ρ				**C. Proportional health-benefit** $\hat{H}$			
	unblended	+P4P	%-change	p-value	unblended	+P4P	%-change	p-value	unblended	+P4P	%-change	p-value
**Fee-For-Service**												
Mild severity of illness												
Illness A	5.77 (2.53)	3.65 (0.52)	-0.37	<0.001	2.81 (2.49)	0.69 (0.47)	-0.75	<0.001	0.60 (0.36)	0.90 (0.07)	0.50	<0.001
Illness B	5.67 (2.55)	3.73 (0.69)	-0.34	<0.001	2.75 (2.46)	0.77 (0.65)	-0.72	<0.001	0.61 (0.35)	0.89 (0.09)	0.47	<0.001
Illness C	5.63 (2.28)	3.71 (0.70)	-0.34	<0.001	2.63 (2.28)	0.74 (0.59)	-0.72	<0.001	0.62 (0.33)	0.89 (0.09)	0.43	<0.001
Interm. severity of illness												
Illness A	6.48 (1.82)	5.60 (0.53)	-0.14	<0.001	1.63 (1.68)	0.63 (0.49)	-0.61	<0.001	0.67 (0.34)	0.87 (0.10)	0.30	<0.001
Illness B	6.87 (1.69)	5.58 (0.54)	-0.19	<0.001	1.90 (1.65)	0.62 (0.49)	-0.67	<0.001	0.62 (0.33)	0.88 (0.10)	0.42	<0.001
Illness C	6.73 (1.73)	5.56 (0.54)	-0.17	<0.001	1.85 (1.60)	0.6 (0.5)	-0.68	<0.001	0.63 (0.32)	0.88 (0.10)	0.40	<0.001
Severe severity of illness												
Illness A	7.69 (1.57)	7.58 (0.64)	-0.01	0.5560	1.08 (1.33)	0.62 (0.6)	-0.43	0.0422	0.85 (0.19)	0.91 (0.09)	0.08	0.0422
Illness B	7.65 (1.37)	7.50 (0.50)	-0.02	0.4757	0.92 (1.20)	0.5 (0.50)	-0.46	0.0388	0.87 (0.17)	0.93 (0.07)	0.07	0.0655
Illness C	7.73 (1.12)	7.42 (0.54)	-0.04	0.0691	0.85 (1.04)	0.46 (0.50)	-0.46	0.0205	0.88 (0.15)	0.93 (0.07)	0.06	0.0205
**Capitation**												
Mild severity of illness												
Illness A	2.07 (1.15)	2.47 (0.69)	0.19	0.0088	0.93 (1.15)	0.6 (0.63)	-0.35	0.0592	0.87 (0.16)	0.91 (0.09)	0.05	0.0592
Illness B	2.20 (1.24)	2.55 (0.72)	0.16	0.1388	0.95 (1.13)	0.56 (0.63)	-0.32	0.0449	0.86 (0.16)	0.92 (0.09)	0.06	0.0717
Illness C	2.42 (1.26)	2.64 (1.04)	0.09	0.6947	0.84 (1.10)	0.62 (0.91)	-0.26	0.2786	0.88 (0.16)	0.91 (0.13)	0.04	0.2786
Interm. severity of illness												
Illness A	3.35 (1.94)	4.36 (0.78)	0.30	<0.001	1.76 (1.84)	0.64 (0.78)	-0.64	<0.001	0.65 (0.37)	0.87 (0.16)	0.35	<0.001
Illness B	3.24 (1.82)	4.40 (0.60)	0.36	<0.001	1.84 (1.74)	0.6 (0.6)	-0.67	<0.001	0.63 (0.35)	0.88 (0.12)	0.39	<0.001
Illness C	3.35 (1.80)	4.38 (0.78)	0.31	<0.001	1.65 (1.70)	0.62 (0.78)	-0.62	<0.001	0.67 (0.34)	0.88 (0.16)	0.31	<0.001
Severe severity of illness												
Illness A	4.27 (2.76)	6.25 (0.78)	0.46	<0.001	2.8 (2.68)	0.78 (0.74)	-0.72	<0.001	0.60 (0.38)	0.89 (0.11)	0.48	<0.001
Illness B	4.25 (2.59)	6.18 (0.98)	0.45	<0.001	2.75 (2.59)	0.82 (0.98)	-0.70	<0.001	0.61 (0.37)	0.88 (0.14)	0.45	<0.001
Illness C	4.60 (2.30)	6.38 (0.62)	0.39	<0.001	2.44 (2.26)	0.65 (0.58)	-0.73	<0.001	0.88 (0.16)	0.91 (0.08)	0.03	<0.001

Notes: This table shows descriptive statistics on the quantities and quality of medical service provision at the level of payment systems, illnesses, severities of illness (means and standard deviations in brackets). 23 non-medical, 22 medical students and 10 physicians decide in the CAP (amounting to a total 990 observations) and 20 non-medical, 22 medical students and 10 physicians in the FFS condition (936 observations). Two-sided p-values are shown for Wilcoxon sigend rank tests for matched samples.

Figure C.1: Distributions of subjects' quantity choice by severity of illness under different payment schemes

(a) CAP

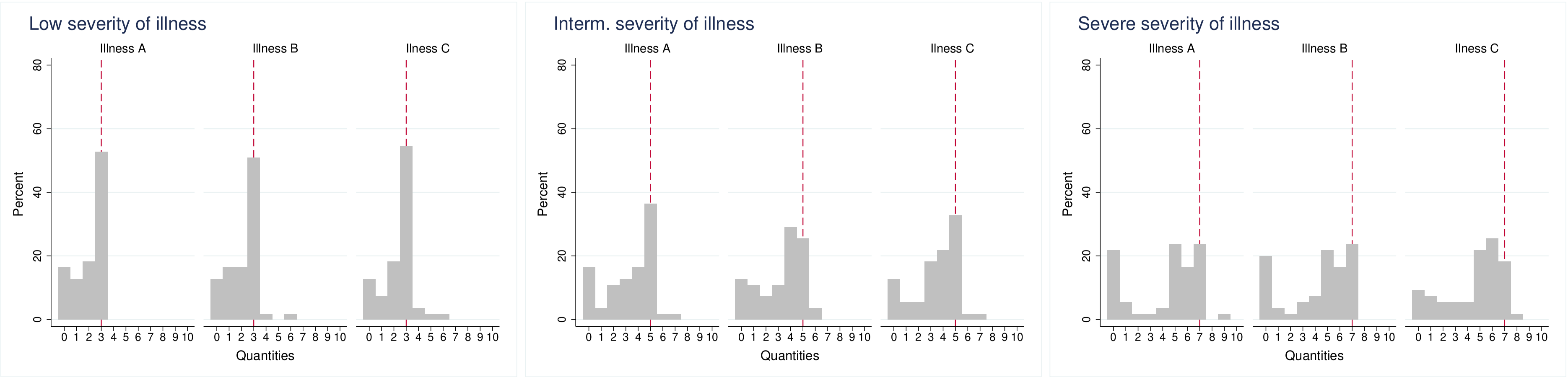

(b) CAP+P4P

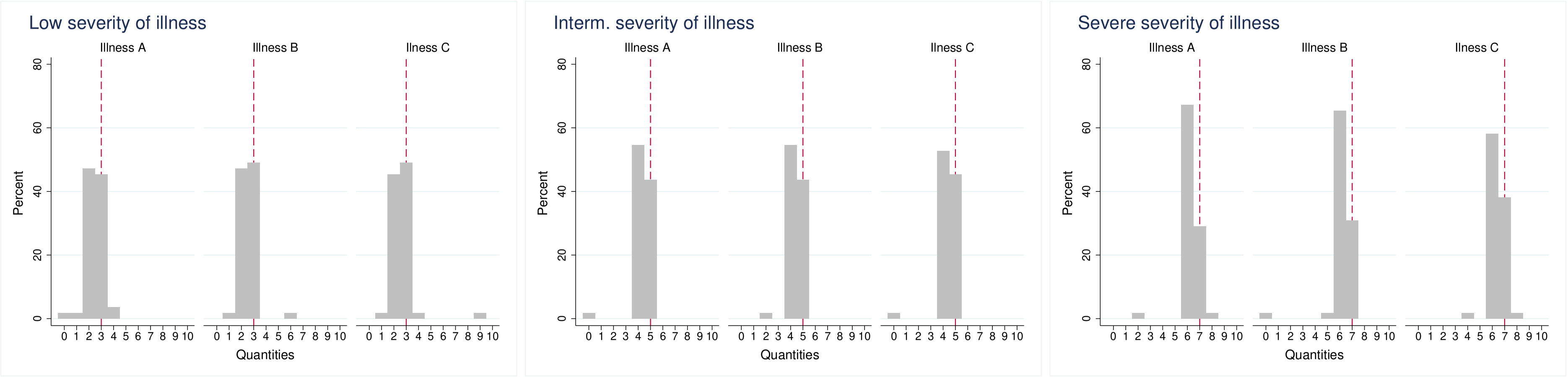

(c) FFS

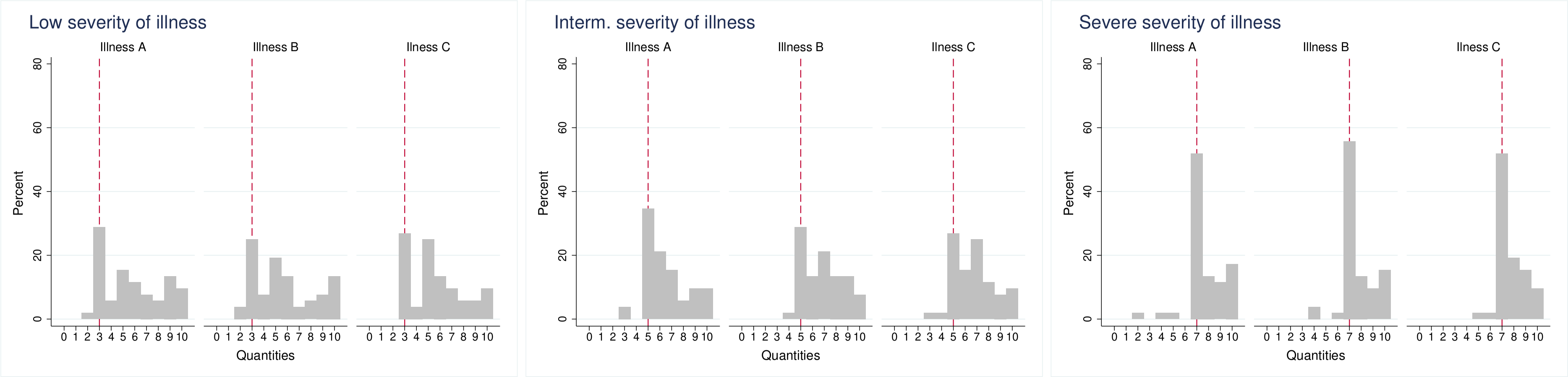

(d) FFS+P4P

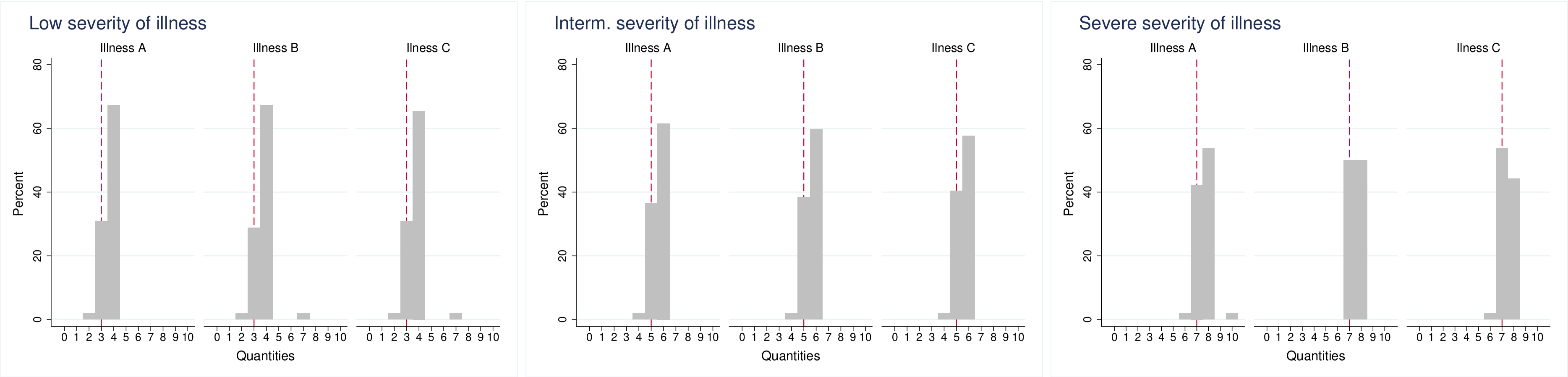

Table C.3: Regression models on the effect on quantity and quality under FFS, without individual controls

	A. Quantity of medical services q			B. Absolute deviation from optimal care ρ			C. Proportional health-benefit $\tilde{H}$		
	(1)	(2)	(3)	(4)	(5)	(6)	(7)	(8)	(9)
P4P	-1.100***			-1.199***			0.191***		
	(0.184)			(0.171)			(0.027)		
IntermSev	1.439***	1.000***	1.439***	-0.529***	-0.936***	-0.529***	0.004	0.020*	0.004
	(0.086)	(0.147)	(0.086)	(0.085)	(0.148)	(0.085)	(0.010)	(0.012)	(0.010)
HighSev	2.901***	2.000***	2.901***	-0.997***	-1.782***	-0.997***	0.136***	0.188***	0.136***
	(0.128)	(0.229)	(0.128)	(0.141)	(0.255)	(0.141)	(0.019)	(0.026)	(0.019)
HighMHB	-0.016	-0.016	0.010	-0.054	-0.054	-0.074	0.008	0.008	0.007
	(0.053)	(0.053)	(0.088)	(0.051)	(0.051)	(0.086)	(0.008)	(0.008)	(0.010)
P4P×MildSev		-1.994***			-1.994***			0.188***	
		(0.276)			(0.276)			(0.024)	
P4P×IntermSev		-1.115***			-1.179***			0.163***	
		(0.191)			(0.182)			(0.023)	
P4P×HighSev		-0.192			-0.423***			0.077***	
		(0.132)			(0.111)			(0.017)	
P4P×LowMHB			-1.083***			-1.212***			0.172***
			(0.195)			(0.179)			(0.025)
P4P×HighMHB			-1.135***			-1.173***			0.154***
			(0.176)			(0.171)			(0.021)
Constant	5.251***	5.698***	5.243***	2.351***	2.749***	2.358***			
	(0.279)	(0.327)	(0.284)	(0.265)	(0.319)	(0.268)			
Wald test (p-value)									
H_0: P4P×MildSev =P4P×IntermSev		<0.001			<0.001			0.010	
H_0: P4P×MildSev= P4P×HighSev		<0.001			<0.001			<0.001	
H_0: P4P×IntermSev=P4P×HighSev		<0.001			<0.001			<0.001	
H_0: P4P×LowMHB=P4P×HighMHB			0.554			0.657			0.056
Observations	936	936	936	936	936	936	936	936	936
Subjects	52	52	52	52	52	52	52	52	52
(Pseudo) R^2	0.449	0.484	0.449	0.219	0.262	0.219	0.091	0.098	0.091

Notes: This table shows parameter estimates from OLS regressions (Panel A and B) and average marginal effects from fractional probit response regressions (Panel C). Robust standard errors clustered for subjects are shown in parentheses. P4P is a dummy variable indicating the introduction of P4P. IntermSev and HighSev are dummy variables for intermediate and high severities of illness. HighMHB is a dummy for the marginal health-benefit being 1 if $\theta = 2$ (high), and 0 otherwise ($\theta = 1$, low).
* $p < 0.10$, ** $p < 0.05$, and, *** $p < 0.01$.

Table C.4: Regression models on the effect on quantity and quality under CAP, without individual controls

	A. Quantity of medical services			B. Abs. deviation from optimal care			C. Proportional health-benefit		
	(1)	(2)	(3)	(4)	(5)	(6)	(7)	(8)	(9)
P4P	1.085***			-1.117***			0.178***		
	(0.188)			(0.179)			(0.029)		
IntermSev	1.473***	1.115***	1.473***	0.436***	0.848***	0.436***	-0.144***	-0.202***	-0.144***
	(0.100)	(0.138)	(0.100)	(0.073)	(0.137)	(0.074)	(0.018)	(0.025)	(0.018)
HighSev	2.933***	2.145***	2.933***	0.958***	1.758***	0.958***	-0.149***	-0.227***	-0.149***
	(0.151)	(0.244)	(0.151)	(0.133)	(0.249)	(0.133)	(0.020)	(0.030)	(0.020)
HighMHB	0.179***	0.179***	0.261***	-0.115**	-0.115**	-0.194**	0.017**	0.017**	0.023***
	(0.048)	(0.048)	(0.069)	(0.044)	(0.044)	(0.073)	(0.007)	(0.007)	(0.009)
P4P×MildSev		0.321**			-0.309***			0.057***	
		(0.140)			(0.108)			(0.018)	
P4P×IntermSev		1.036***			-1.133***			0.158***	
		(0.200)			(0.188)			(0.024)	
P4P×HighSev		1.897***			-1.909***			0.182***	
		(0.295)			(0.291)			(0.026)	
P4P×LowMHB			1.139***			-1.170***			0.166***
			(0.195)			(0.188)			(0.026)
P4P×HighMHB			0.976***			-1.012***			0.137***
			(0.192)			(0.172)			(0.021)
Constant	1.789***	2.171***	1.762***	1.345***	0.941***	1.372***			
	(0.180)	(0.147)	(0.183)	(0.173)	(0.133)	(0.178)			
Wald test (p-value)									
H_0: P4P×MildSev =P4P×IntermSev		<0.001			<0.001			<0.001	
H_0: P4P×MildSev= P4P×HighSev		<0.001			<0.001			<0.001	
H_0: P4P×IntermSev=P4P×HighSev		<0.001			<0.001			0.011	
H_0: P4P×LowMHB=P4P×HighMHB			0.096			0.048			0.004
Observations	990	990	990	990	990	990	990	990	990
Subjects	55	55	55	55	55	55	55	55	55
(Pseudo) R^2	0.432	0.457	0.432	0.180	0.221	0.180	0.082	0.090	0.082

Notes: This table shows parameter estimates from OLS regressions (Panel A and B) and average marginal effects from fractional probit response regressions (Panel C). Robust standard errors clustered for subjects are shown in parentheses. P4P is a dummy variable indicating the introduction of P4P. IntermSev and HighSev are dummy variables for intermediate and high severities of illness. HighMHB is a dummy for the marginal health-benefit being 1 if $\theta = 2$ (high), and 0 otherwise ($\theta = 1$, low).
* $p < 0.10$, ** $p < 0.05$, and, *** $p < 0.01$.

Table C.5: Regression models on the effect on quantity and quality under FFS conditions with the full list of covariates

	A. Quantity of medical services q			B. Absolute deviation from optimal care ρ			C. Proportional health-benefit $\hat{H}$		
Method:	OLS	OLS	OLS	OLS	OLS	OLS	Frac. Probit	Frac. Probit	Frac. Probit
Model:	(1)	(2)	(3)	(4)	(5)	(6)	(7)	(8)	(9)
P4P	-1.100***			-1.199***			0.189***		
	(0.185)			(0.172)			(0.025)		
IntermSev	1.439***	1.000***	1.439***	-0.529***	-0.936***	-0.529***	0.004	0.019	0.004
	(0.086)	(0.147)	(0.086)	(0.086)	(0.149)	(0.086)	(0.010)	(0.012)	(0.010)
HighSev	2.901***	2.000***	2.901***	-0.997***	-1.782***	0.997***	0.133***	0.184***	0.133***
	(0.129)	(0.230)	(0.129)	(0.141)	(0.256)	(0.141)	(0.016)	(0.023)	(0.016)
HighMHB	-0.016	-0.016	0.010	-0.054	-0.054	-0.074	0.009	0.009	0.008
	(0.053)	(0.053)	(0.089)	(0.051)	(0.051)	(0.087)	(0.008)	(0.008)	(0.011)
P4P×MildSev		-1.994***			-1.994***			0.187***	
		(0.277)			(0.277)			(0.021)	
P4P×IntermSev		-1.115***			-1.179***			0.162***	
		(0.192)			(0.183)			(0.021)	
P4P×HighSev		-0.192			-0.423***			0.079***	
		(0.132)			(0.111)			(0.017)	
P4P×LowMHB			-1.083***			-1.212***			0.171***
			(0.195)			(0.179)			(0.022)
P4P×HighMHB			-1.135***			-1.173***			0.153***
			(0.177)			(0.172)			(0.018)
Medical students	-0.402	-0.402	-0.402	-0.388	-0.388	-0.388	0.064	0.063	0.064
	(0.306)	(0.307)	(0.307)	(0.299)	(0.299)	(0.299)	(0.051)	(0.051)	(0.051)
Physicians	-1.909***	-1.909***	-1.909***	-1.520***	-1.520***	-1.520***	0.230***	0.228***	0.230***
	(0.270)	(0.271)	(0.270)	(0.266)	(0.267)	(0.267)	(0.041)	(0.040)	(0.041)
Male	0.043	0.043	0.043	0.050	0.050	0.050	-0.002	-0.002	-0.002
	(0.223)	(0.223)	(0.223)	(0.222)	(0.222)	(0.222)	(0.035)	(0.035)	(0.035)
Extraversion	0.227	0.227	0.227	0.275	0.275	0.275	-0.042	-0.042	-0.042
	(0.286)	(0.286)	(0.286)	(0.298)	(0.299)	(0.299)	(0.045)	(0.045)	(0.045)
Neuroticism	0.037	0.037	0.037	0.274	0.274	0.274	-0.046	-0.046	-0.046
	(0.255)	(0.255)	(0.255)	(0.258)	(0.258)	(0.258)	(0.042)	(0.042)	(0.042)
Openness	-0.151	-0.151	-0.151	-0.021	-0.021	-0.021	0.003	0.004	0.003
	(0.287)	(0.287)	(0.287)	(0.292)	(0.293)	(0.292)	(0.044)	(0.044)	(0.044)
Conscientiousness	0.477	0.477	0.477	0.428	0.428	0.428	-0.065	-0.065	-0.065
	(0.316)	(0.317)	(0.316)	(0.325)	(0.326)	(0.326)	(0.051)	(0.051)	(0.051)
Agreeableness	0.097	0.097	0.097	0.136	0.136	0.136	-0.030	-0.030	-0.030
	(0.306)	(0.307)	(0.306)	(0.315)	(0.315)	(0.315)	(0.053)	(0.053)	(0.053)
Constant	5.623***	6.070***	5.615***	2.621***	3.019***	2.627***			
	(0.315)	(0.350)	(0.318)	(0.315)	(0.354)	(0.317)			
Wald test (p-value)									
H_0: P4P×MildSev =P4P×IntermSev		<0.001			<0.001			0.010	
H_0: P4P×MildSev= P4P×HighSev		<0.001			<0.001			<0.001	
H_0: P4P×IntermSev=P4P×HighSev		<0.001			<0.001			<0.001	
H_0: P4P×LowMHB=P4P×HighMHB			0.556			0.658			0.062
Observations	936	936	936	936	936	936	936	936	936
Subjects	52	52	52	52	52	52	52	52	52
(Pseudo) R^2	0.563	0.599	0.563	0.336	0.379	0.336	0.150	0.157	0.150

Notes: This table shows parameter estimates from OLS regressions (Panel A and B) and average marginal effects from fractional probit response regressions (Panel C). Robust standard errors clustered for subjects are shown in parentheses. P4P is a dummy variable indicating the introduction of P4P. IntermSev and HighSev are dummy variables for intermediate and high severities of illness. HighMHB is a dummy for the marginal health-benefit being 1 if $\theta = 2$ (high), and 0 otherwise ($\theta = 1$, low). All models control for individual characteristics which comprise gender, medical background (non-medical student, medical student, physician), and personality traits.
* $p < 0.10$, ** $p < 0.05$, and, *** $p < 0.01$.

Table C.6: Regression models on the effect on quantity and quality under CAP conditions with the full list of covariates

	A. Quantity of medical services q			B. Absolute deviation from optimal care ρ			C. Proportional health-benefit $\hat{H}$		
Method:	OLS	OLS	OLS	OLS	OLS	OLS	Frac. Probit	Frac. Probit	Frac. Probit
Model:	(1)	(2)	(3)	(4)	(5)	(6)	(7)	(8)	(9)
P4P	1.085***			-1.117***			0.175***		
	(0.189)			(0.180)			(0.026)		
IntermSev	1.473***	1.115***	1.473***	0.436***	0.848***	0.436***	-0.143***	-0.201***	-0.143***
	(0.100)	(0.139)	(0.100)	(0.074)	(0.138)	(0.074)	(0.016)	(0.024)	(0.016)
HighSev	2.933***	2.145***	2.933***	0.958***	1.758***	0.958***	-0.149***	-0.227***	-0.149***
	(0.151)	(0.245)	(0.151)	(0.134)	(0.250)	(0.134)	(0.019)	(0.028)	(0.019)
HighMHB	0.179***	0.179***	0.261***	-0.115**	0.115**	-0.194**	0.017**	0.017**	0.024***
	(0.048)	(0.048)	(0.069)	(0.044)	(0.044)	(0.073)	(0.007)	(0.007)	(0.009)
P4P×MildSev		0.321**			-0.309***			0.055***	
		(0.140)			(0.108)			(0.017)	
P4P×IntermSev		1.036***			-1.133***			0.157***	
		(0.201)			(0.189)			(0.021)	
P4P×HighSev		1.897***			-1.909***			0.180***	
		(0.296)			(0.292)			(0.023)	
P4P×LowMHB			1.139***			-1.170***			0.165***
			(0.195)			(0.188)			(0.024)
P4P×HighMHB			0.976***			-1.012***			0.135***
			(0.193)			(0.173)			(0.018)
Medical students	-0.165	-0.165	-0.165	0.192	0.192	0.192	-0.038	-0.039	-0.038
	(0.281)	(0.281)	(0.281)	(0.280)	(0.281)	(0.281)	(0.046)	(0.046)	(0.046)
Physicians	0.497	0.497	0.497	-0.438	-0.438	-0.438	0.073	0.072	0.073
	(0.324)	(0.324)	(0.324)	(0.322)	(0.323)	(0.323)	(0.048)	(0.048)	(0.048)
Male	-0.436*	-0.436*	-0.436*	0.385	0.385	0.385	-0.057	-0.057	-0.057
	(0.258)	(0.258)	(0.258)	(0.263)	(0.263)	(0.263)	(0.045)	(0.045)	(0.045)
Extraversion	0.497*	0.497*	0.497*	-0.430	-0.430	-0.430	0.059	0.059	0.059
	(0.283)	(0.283)	(0.283)	(0.296)	(0.296)	(0.296)	(0.045)	(0.045)	(0.045)
Neuroticism	0.354	0.354	0.354	-0.301	-0.301	-0.301	0.048	0.047	0.048
	(0.213)	(0.213)	(0.213)	(0.226)	(0.226)	(0.226)	(0.041)	(0.040)	(0.041)
Openness	-0.257	-0.257	-0.257	0.198	0.198	0.198	-0.036	-0.035	-0.036
	(0.237)	(0.237)	(0.237)	(0.252)	(0.252)	(0.252)	(0.039)	(0.039)	(0.039)
Conscientiousness	0.622**	0.622**	0.622**	-0.690**	-0.690**	-0.690**	0.098**	0.097**	0.098**
	(0.305)	(0.305)	(0.305)	(0.305)	(0.305)	(0.305)	(0.047)	(0.046)	(0.047)
Agreeableness	0.874***	0.874***	0.874***	-0.766**	-0.766**	-0.766**	0.121**	0.120**	0.121**
	(0.300)	(0.300)	(0.300)	(0.297)	(0.297)	(0.297)	(0.048)	(0.048)	(0.048)
Constant	1.725***	2.107***	1.698***	1.440***	1.036***	1.466***			
	(0.250)	(0.231)	(0.254)	(0.242)	(0.227)	(0.247)			
Wald test (p-value)									
H_0: P4P×MildSev=P4P×IntermSev		<0.001			<0.001			<0.001	
H_0: P4P×MildSev=P4P×HighSev		<0.001			<0.001			<0.001	
H_0: P4P×IntermSev=P4P×HighSev		<0.001			<0.001			0.015	
H_0: P4P×LowMHB=P4P×HighMHB			0.097			0.049			0.004
Observations	990	990	990	990	990	990	990	990	990
Subjects	55	55	55	55	55	55	55	55	55
(Pseudo) R^2	0.509	0.534	0.509	0.287	0.328	0.287	0.131	0.140	0.131

Notes: This table shows parameter estimates from OLS regressions (Panel A and B) and average marginal effects from fractional probit response regressions (Panel C). Robust standard errors clustered for subjects are shown in parentheses. P4P is a dummy variable indicating the introduction of P4P. IntermSev and HighSev are dummy variables for intermediate and high severities of illness. HighMHB is a dummy for the marginal health-benefit being 1 if $\theta = 2$ (high), and 0 otherwise ($\theta = 1$, low). All models control for individual characteristics which comprise gender, medical background (non-medical student, medical student, physician), and personality traits.
* $p < 0.10$, ** $p < 0.05$, and, *** $p < 0.01$.

Table C.7: Comparison of effects when introducing performance pay to fee-for-service and capitation, with full list of covariates

	A. Absolute deviation from patient-optimal care ρ		B. Proportional health-benefit $\hat{H}$	
Method:	OLS	OLS	Frac. Probit	Frac. Probit
Model:	(1)	(2)	(3)	(4)
CAP	-1.828***	-1.832***	0.210***	0.209***
	(0.342)	(0.310)	(0.037)	(0.032)
IntermSev	-0.936***	-0.936***	0.020*	0.020
	(0.147)	(0.148)	(0.012)	(0.012)
HighSev	-1.782***	-1.782***	0.182***	0.178***
	(0.253)	(0.254)	(0.021)	(0.020)
HighMHB	-0.086**	-0.086**	0.013**	0.013**
	(0.033)	(0.034)	(0.005)	(0.005)
CAP×IntermSev	1.784***	1.784***	-0.243***	-0.241***
	(0.201)	(0.201)	(0.029)	(0.028)
CAP×HighSev	3.540***	3.540***	-0.492***	-0.484***
	(0.354)	(0.355)	(0.033)	(0.033)
CAP+P4P×MildSev	-0.309***	-0.309***	0.056***	0.054***
	(0.107)	(0.107)	(0.017)	(0.016)
FFS+P4P×MildSev	-1.994***	-1.994***	0.171***	0.170***
	(0.274)	(0.275)	(0.017)	(0.016)
CAP+P4P×IntermSev	-1.133***	-1.133***	0.148***	0.148***
	(0.187)	(0.188)	(0.018)	(0.017)
FFS+P4P×IntermSev	-1.179***	-1.179***	0.151***	0.150***
	(0.181)	(0.182)	(0.017)	(0.016)
CAP+P4P×HighSev	-1.909***	-1.909***	0.168***	0.167***
	(0.289)	(0.290)	(0.018)	(0.017)
FFS+P4P×HighSev	-0.423***	-0.423***	0.075***	0.077***
	(0.110)	(0.111)	(0.015)	(0.015)
Medical students		-0.136		0.015
		(0.215)		(0.035)
Physicians		-0.894***		0.142***
		(0.233)		(0.033)
Male		0.185		-0.031
		(0.189)		(0.030)
Extraversion		0.008		-0.004
		(0.214)		(0.032)
Neuroticism		-0.051		0.005
		(0.195)		(0.033)
Openness		0.078		-0.016
		(0.198)		(0.030)
Conscientiousness		-0.199		0.029
		(0.253)		(0.037)
Agreeableness		-0.470**		0.078**
		(0.236)		(0.037)
Constant	2.759***	2.950***		
	(0.316)	(0.324)		
Individual controls	No	Yes	No	Yes
Wald tests (p-value):				
H_0: CAP+P4P×MildSev = FFS+P4P×MildSev	$<$0.001	$<$0.001	$<$0.001	$<$0.001
H_0: CAP+P4P×IntermSev = FFS+P4P×IntermSev	0.860	0.860	0.872	0.884
H_0: CAP+P4P×HighSev = FFS+P4P×HighSev	$<$0.001	$<$0.001	$<$0.001	$<$0.001
Observations	1926	1926	1926	1926
Subjects	107	107	107	107
(Pseudo) R^2	0.240	0.312	0.094	0.129

Notes: For Panel A OLS estimates are reported with robust standard errors clustered for subjects (in brackets). For Panel B average marginal effects (AMEs) based on a fractional probit response are reported with robust standard errors clustered for subjects (in brackets). CAP = 1 if physicians are remunerated by CAP, and = 0 otherwise (by FFS). P4P is a dummy variable indicating the introduction of P4P. IntermSev and HighSev are dummy variables for intermediate and high severities of illness. HighMHB is a dummy for the marginal health-benefit being 1 if θ = 2 (high), and 0 otherwise (θ = 1, low). Controls for subjects' individual characteristics comprise gender, medical background (non-medical student, medical student, physician), and personality traits. * $p < 0.10$, ** $p < 0.05$, and *** $p < 0.01$.

References

Brosig-Koch, J., Hennig-Schmidt, H., Kairies-Schwarz, N., and Wiesen, D. (2016). Using artefactual field and lab experiments to investigate how fee-for-service and capitation affect medical service provision. *Journal of Economic Behavior & Organization*, 131, Part B:17–23.

Brosig-Koch, J., Hennig-Schmidt, H., Kairies-Schwarz, N., and Wiesen, D. (2017). The effects of introducing mixed payment systems for physicians: Experimental evidence. *Health Economics*, 26:243 – 262.

Hennig-Schmidt, H., Selten, R., and Wiesen, D. (2011). How payment systems affect physicians' provision behavior – An experimental investigation. *Journal of Health Economics*, 30:637–646.

Rammstedt, B. and John, O. P. (2007). Measuring personality in one minute or less: A 10-item short version of the big five inventory in English and German. *Journal of Research in Personality*, 41:203–212.
